# Gate tunable giant anisotropic resistance in ultra-thin GaTe

**DOI:** 10.1038/s41467-019-10256-3

**Published:** 2019-05-24

**Authors:** Hanwen Wang, Mao-Lin Chen, Mengjian Zhu, Yaning Wang, Baojuan Dong, Xingdan Sun, Xiaorong Zhang, Shimin Cao, Xiaoxi Li, Jianqi Huang, Lei Zhang, Weilai Liu, Dongming Sun, Yu Ye, Kepeng Song, Jianjian Wang, Yu Han, Teng Yang, Huaihong Guo, Chengbing Qin, Liantuan Xiao, Jing Zhang, Jianhao Chen, Zheng Han, Zhidong Zhang

**Affiliations:** 1 Shenyang National Laboratory for Materials Science, Institute of Metal Research, Chinese Academy of Sciences, Shenyang, 110016 China; 2 School of Material Science and Engineering, University of Science and Technology of China, Anhui, 230026 China; 3 College of Advanced Interdisciplinary Studies, National University of Defense Technology, Changsha, 410073 China; 4 State Key Laboratory of Quantum Optics and Quantum Optics Devices, Institute of Laser Spectroscopy, Shanxi University, Taiyuan, 030006 China; 5 Collaborative Innovation Center of Extreme Optics, Shanxi University, Taiyuan, 030006 China; 6 International Center for Quantum Materials, School of Physics, Peking University, Beijing, 100871 China; 7 Collaborative Innovation Center of Quantum Matter, Beijing, 100871 China; 8 State Key Lab for Mesoscopic Physics and School of Physics, Peking University, Beijing, China; 9 Advanced Membranes and Porous Materials Center, Physical Science and Engineering Division, King Abdullah University of Science and Technology, Thuwal, 23966-6900 Saudi Arabia; 10 Multi-scale Porous Materials Center, Institute of Advanced Interdisciplinary Studies, Chongqing University, Chongqing, 400044 China; 11 College of Sciences, Liaoning Shihua University, Fushun, 113001 China; 12 State Key Laboratory of Quantum Optics and Quantum Optics Devices, Institute of Opto-Electronics, Shanxi University, Taiyuan, 030006 China

**Keywords:** Electronic devices, Two-dimensional materials

## Abstract

Anisotropy in crystals arises from different lattice periodicity along different crystallographic directions, and is usually more pronounced in two dimensional (2D) materials. Indeed, in the emerging 2D materials, electrical anisotropy has been one of the recent research focuses. However, key understandings of the in-plane anisotropic resistance in low-symmetry 2D materials, as well as demonstrations of model devices taking advantage of it, have proven difficult. Here, we show that, in few-layered semiconducting GaTe, electrical conductivity anisotropy between **x** and **y** directions of the 2D crystal can be gate tuned from several fold to over 10^3^. This effect is further demonstrated to yield an anisotropic non-volatile memory behavior in ultra-thin GaTe, when equipped with an architecture of van der Waals floating gate. Our findings of gate-tunable giant anisotropic resistance effect pave the way for potential applications in nanoelectronics such as multifunctional directional memories in the 2D limit.

## Abstract (web summary)

Some atomically thin crystals feature crystallographic anisotropy, but demonstrations of electrical anisotropy are scarce. Here, the authors show that the electrical conductivity of few-layered GaTe along the **x** and **y** directions can be widely gate tuned up to 10^3^, and demonstrate anisotropic non-volatile memory behavior.

## Introduction

It is known that when electrically measuring a bulk material, the resulted conductivity may manifest strong directional dependencies^1,2^. Discrepancy of conductivity along different in-plane directions in layered bulk crystals can sometimes be as large as a few hundreds, which, however, often requires a certain conditions such as the presence of large external magnetic field^3^. Recently, low-symmetry 2D materials have attracted significant attentions because of the potential applications of in-plane anisotropic nanoelectronics^4–11^. For example, ultra-thin black phosphorous flake showed an in-plane anisotropic conductance reaching a ratio *σ*_a_/*σ*_b_ of ~ 1.5, which in principle can be a direction-sensitive sensor^4^. ReS_2_ was reported to be another candidate for anisotropic 2D field effect transistor, which exhibited a *σ*_a_ of almost 10 times the value of *σ*_b_^9^. Recent studies on GeP and GeAs flakes also showed anisotropic behavior with anisotropic factors of 1.5~2 for their conductance^10,11^.

**Fig. 1:**
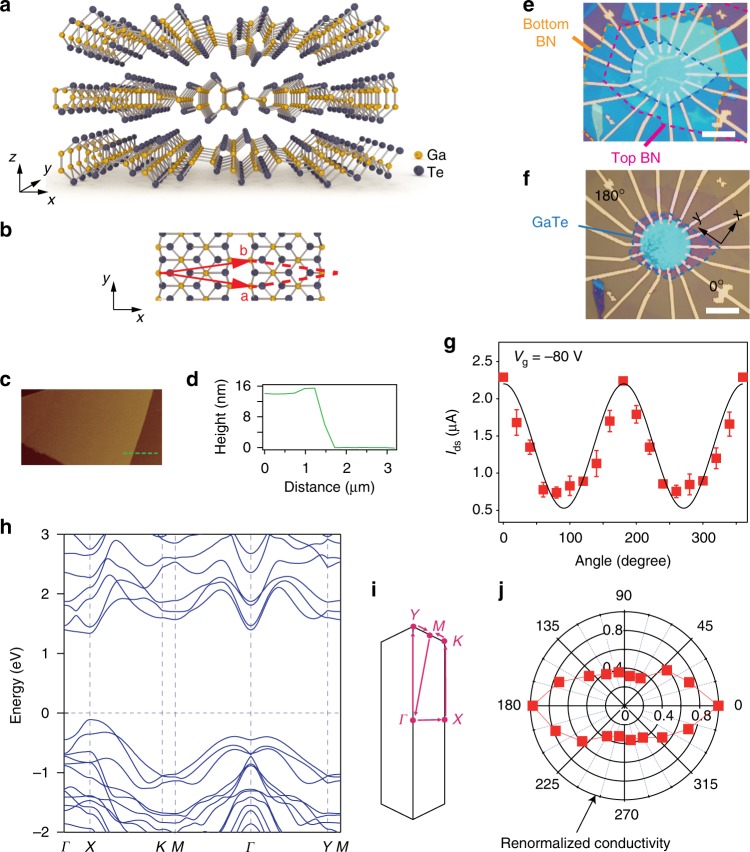
Characterizations of ultra-thin GaTe. **a** Schematics of GaTe crystal structure, with its in-plane unit cell illustrated in **b**. **c** AFM image of a typical GaTe flake of ~ 14 nm in thickness, with its height profile along the green dashed line plotted in **d**. **e** Optical image of a typical device (sample S1) made of 14 nm GaTe flake encapsulated in h-BN. Electrodes are patterned with an angle interval of 20 degree. **f** Same device in **e** but patterned into a circular shape. Scale bars in **e** and **f** are 10 μm. **g** Source-drain current at *V*_ds_ = 2 V as a function of angle, with *V*_g_ = −80 V. The black solid line is an ellipsoidal fit. Error bars in **g** are defined as the deviations between experimental data and the fitted line. **h** Electronic band structure of monolayer GaTe, with the first Brillouin zone plotted in **i**. **j** Same data in **g** plotted after re-normalization in a polar graph

To date, in-plane anisotropic factor *Γ*_a_ of electrical conductance in 2D materials is yet limited within one order of magnitude under ambient condition. It thus hinders future applications owing to a weak effective conductance difference between directions, and a larger anisotropic factor is highly desired. In this work, we show an observation of giant anisotropic resistance (GAR) behavior in few-layered *p*-type semiconducting GaTe. Among devices measured, the electrical conductivity ratio along *x* and *y* (*x* and *y* are defined in Fig. 1) directions of ultra-thin GaTe reaches an order of 10^3^ at gate voltages close to the valence-band maximum (VBM), which can be further gate tuned down to < 10, upon hole doping. We show detailed analysis and physical understandings on the GAR effect. Based on this, floating gate anisotropic memory with directional multi-level outputs are demonstrated. Moreover, when measuring along fixed direction, the few-layered GaTe floating gate memory (FGM) exhibits On/Off ratio of 10^7^ and retention time of 10^5^ s, which is by far the best performance among FGMs made of 2D materials. Our findings open new possibilities toward next generation nanoelectronics, such as artificial neuron network based on anisotropic memory arrays.

## Results

### Characterizations of ultra-thin GaTe

Bulk GaTe single crystals were prepared via flux method and were confirmed via x-ray diffraction (see Supplementary Fig. 1). We mechanically exfoliated the bulk and deposited ultra-thin GaTe flakes onto 285 nm silicon oxide grown on heavily doped silicon wafers for optical and electrical characterizations. It is known that GaTe has a layered structure with lattice symmetry of group *C*_2h_ (Fig. 1a), similar to that of 1 T′-MoTe_2_. The unit cell for a single layer GaTe is indicated in Fig. 1b. Monoclinic GaTe crystal is of very low symmetry, providing an ideal platform to study anisotropy^12^. It is also known as a typical p-type semiconductor with a direct band gap of ~ 1.65 eV^13,14^, which has many potential applications, such as photodetectors^15^, radiation detectors^16^, as well as thermophotovoltaic devices^17^. However, GaTe in air was reported to be easily oxidized^18^, leading to band gap restructuring in air, at the same time, the photoluminescence (PL) signal decreases and extra Raman peaks associated to oxides become more pronounced. Atomic Force Microscope (AFM) scan of a typical GaTe flake (14 nm in thickness) is shown in Fig. 1c, d. First, we characterized electronic transport of those flakes in air (Supplementary Figs. 2–6). However, without protection from air, the source-drain currents *I*_ds_ of devices made by GaTe flakes are rather low, with maximum current of a few nano amperes, in agreement with other reported results^19^. It is found that, without thermal annealing, few-layered GaTe devices are barely conducting, as shown in Supplementary Fig. 2. In the following, we will focus on the devices made by ultra-thin GaTe flakes encapsulated by hexagonal boron nitride (h-BN) in a glove box, in order to diminish damages from air. Detailed analysis of the effect of h-BN protection can be found in Supplementary Figs. 7–11.

Polarized Raman can be used to determine crystal orientation in 2D flakes^10,12^, and also to deliver information of phonon modes that are related to the lattice symmetry. Supplementary Fig. 12 shows Raman spectra of a typical GaTe flake, with the angle-dependent intensity at Raman shift of 271.1 cm^−1^ plotted in a polar figure overlaid on the optical image shown in the inset. A twofold symmetry of the polar figure can be seen, with one of the polarized axis parallel to the exfoliated straight edge of the GaTe itself. Such a Raman-active mode is a *A*_g_ mode in *C*_2h_ point group. From Raman tensor analysis, the direction of maximal Raman intensity of such *A*_g_ mode corresponds to the lattice direction with short lattice constant, defined as *y* direction in Fig. 1a, with *x* direction perpendicular to it. Raman mode at 164.6 cm^−1^ is another good reference for determining the crystalline direction (see Supplementary Fig. 13)^12^. Such a mode has a fourfold symmetry (*B*_g_ mode) and therefore four-lope angular dependence, with each lope 45 degrees away from the polarization axis of the mode at 271.1 cm^−1^. By comparing Raman anisotropy with the anisotropy of electrical conductivity (shown in the next section), it is known that *y* axis of GaTe layer corresponds to a maximum conductivity (Supplementary Figs. 12–13). By this means, one can pattern electrodes for electrical measurements just with the zero angle defined along with such straight edges (*y* direction).

As shown in Fig. 1e, nine pairs of electrodes (20° angle between each two electrodes) were deposited onto the h-BN/GaTe/h-BN stack sample S1 (see Methods), which is further patterned into a circular shape in Fig. 1f. As a result, the source-drain current *I*_ds_ along each pair of electrodes at gate voltage *V*_g_ = −80 V follows an ellipsoidal dependence of the testing angle, shown in Fig. 1g. It is rather straight forward that the maximum *I*_ds_ flows in 0°, which is parallel to one of the straight edges, i.e., the *y* axis, as marked in Fig. 1f. Such twofold ellipsoidal oscillation of in-plane anisotropic conductivity is also seen in a number of other 2D materials^4,5,9–11,20^.

To unveil the origin of in-plane electrical conductivity anisotropy found in ultra-thin GaTe, we performed first-principles electronic structure and non-equilibrium Green’s functional quantum transport calculations with a simplified model on GaTe monolayer. Electronic band structure along high-symmetry lines is shown in Fig. 1h, with the first Brillouin zone and high-symmetry points defined in Fig. 1i. A direct band gap of 1.5 eV locates at the *X* point, in agreement with PL measurement in Supplementary Fig. 14. Thanks to the direct band gap, outstanding photo responses have been reported in GaTe devices^15,21^. What’s very interesting is the strongly anisotropic band dispersion obviously seen along two perpendicular directions (*Γ–X* and *X–K*). At the VBM, band dispersion along *Γ–X* is much bigger than along *X–K*, which gives rise to strong anisotropic effective mass m^*^ at VBM, as shown in Supplementary Fig. 15. The calculated m^*^ (shown in Supplementary Fig. 16) along *X–K* direction is ~ 10 times larger than that along *Γ–X*, which seems to lead to a better transport property along *x* axis than *y* axis. However, deformation potential *E*_1_ (shown in Supplementary Fig. 16) due to electron-phonon scattering shows an opposite trend to m^*^ in both *x* and *y* axes, and dominates over the anisotropy of effective mass to give rise to the anisotropy of electrical conductivity *σ* according to the deformation potential theory (see Methods), *σ*_y_ is about five times as big as *σ*_x_ at low doping. When plotting experimental data in the renormalized conductivity polar figure in Fig. 1j, a twofold symmetry of conductance can be clearly seen.

### Gate-tunable GAR in ultra-thin GaTe

**Fig. 2:**
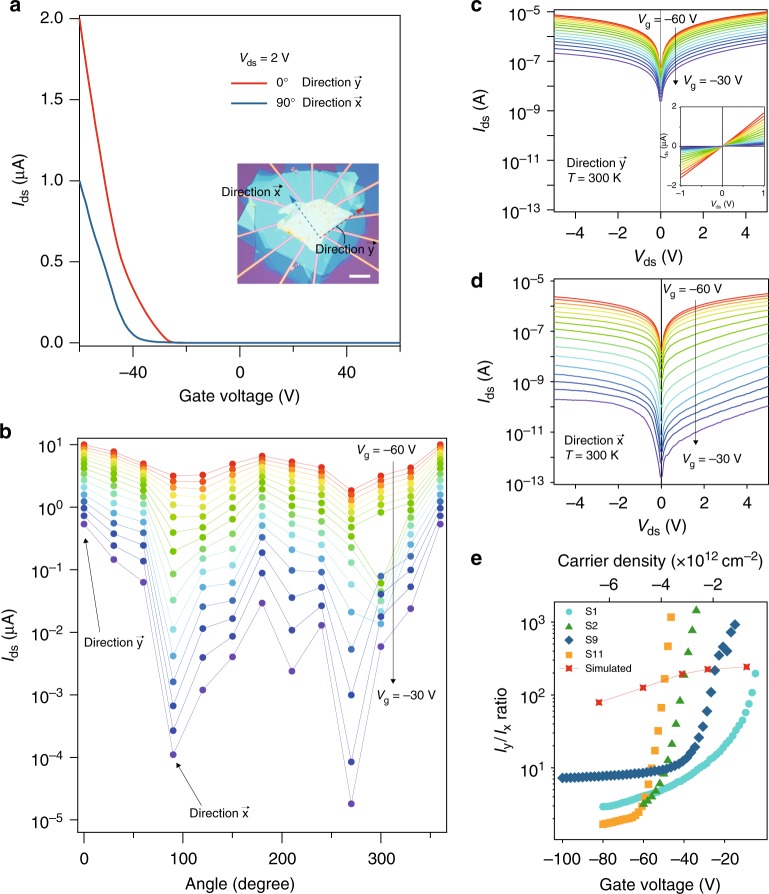
Giant anisotropic resistance in ultra-thin GaTe. **a** Field effect curves of a typical GaTe device (Sample S2) recorded along *x* and *y* directions, with the optical image of the device shown in the inset. Scale bar in the inset is 10 μm. **b** *I*_ds_ of the same device measured along the 12 directions with a fixed *V*_ds_ = 5 V, and with the gate voltages swept from −60 V to −30 V. **c**, **d** *I–V* curves plotted in log scale at *V*_ds_ = ±5 V of the same device measured along *y* and *x* directions, respectively. The gate voltages were swept from −60 V to −30 V. Inset of **c** shows the corresponding data in linear scale. **e** The maximal electrical anisotropic ratio *I*_y_/*I*_x_ extracted from different samples (denoted as S1, S2, S9, and S11) as a function of gate voltage

The above-mentioned twofold in-plane symmetry of resistivity can be reproduced in multiple GaTe devices (Supplementary Figs. 17–18), and we performed a systematic study on the sample S2. As shown in Fig. 2a, with the same GaTe flake (optical image of the device is shown in the inset of Fig. 2a), field–effect curves, measured at source-drain voltage *V*_ds_ = 2 V along *x* and *y* directions, exhibit strong anisotropy, indicated by red and blue colors, respectively. *I*_ds_ measured along the 12 directions with a fixed *V*_ds_ = 5 V are plotted at *V*_g_ = −60 V, while an ellipsoidal oscillation at all angles with 2*π* periodicity can be seen, shown in Fig. 2b. When the gate voltages were swept from −60 V to −30 V, the maximum-to-minimum *I*_ds_ ratio of the ellipsoidal oscillation at each gate voltage varies largely. Note that similar gate-tunable anisotropic resistance behavior is also seen in bare GaTe devices without BN encapsulation (Supplementary Figs. 19–20).

Taking *y* (0°) and *x* (90°) directions for examples, closer look of *I–V* output curves in the range of ±5 V is shown in Fig. 2c–d. It is seen that at high bias voltage, *I*_ds_ for direction *y* is varying within 10^−7^ and 10^−5^ A, whereas *I*_ds_ for direction *x* is varying within 10^−11^ and 10^−6^ A. We define the electrical maximum anisotropic ratio *Γ*_a_ as *I*_y_/*I*_x_ for each fixed gate voltage. Strikingly, as extracted from Fig. 2b, *Γ*_a_ as a function of gate voltage shows a gate-tunability from less than one order to as large as 10^3^. This gate-tunable GAR effect was not found in previous studies. We fabricated various samples in similar configuration of h-BN encapsulated ultra-thin GaTe devices (Supplementary Figs. 21–23), with their *Γ*_a_ plotted together in Fig. 2e. In general, anisotropic ratio in those devices can be gate tuned from a few times, to several hundreds or even thousands folds, which is much higher than other 2D systems reported so far^4,5,7,9,22^. To push the 2D limit of the GaTe thicknesses studied in this work, we managed to fabricate sample of GaTe with ~ 4.8 nm in thickness. And as the inter-layer distance of GaTe is ~ 0.8 nm^21^, we can draw the conclusion that, down to the six-layer limit, the observed GAR prevails, as shown in Supplementary Fig. 24.

As *Γ*_a_ in ultra-thin GaTe is rather weak at relatively higher hole doping (say, in the *V*_g_ = −60 V limit), and becomes significantly enhanced close to the VBM in the vicinity of *V*_g_ = −30 V, it more or less shows a better electron collimation at higher *Γ*_a_ values, i.e., electrons tend to flow preferably along a certain direction at those conditions. Considering the deformation potential theory only applied to the band edge (VBM or CBM) and its inability to take care the gate-tunability on electronic transport^23,24^, we calculate *I–V* curves and their gate dependence by combining density functional theory (DFT) calculation with the non-equilibrium Green’s function (NEGF) method^25^ (see Methods), and show calculated field effect I-V data at *V*_ds_ = 0.5 V along two perpendicular crystalline directions in Supplementary Fig. 25. To avoid possible contact Schottky barrier, we use *p*-type heavily doped GaTe as source and drain. *I–V* curves along both *x* and *y* directions show a similar behavior to the experimental ones as given in Fig. 2a. *I*_y,ds_ at *V*_ds_ = 0.5 V increases from 1.39 μA at *V*_g_ = −9.1 V to 12.52 μA at *V*_g_ = −82 V. More strikingly is the gate dependence of *I*_x,ds_, which changes by two orders of magnitude, from 4.63 × 10^−3^ μA at *V*_g_ = −9.1 V to 0.19 μA at *V*_g_ = −82 V. The calculated *I*_y_/*I*_x_ ratio, given in filled red squares in Fig. 2e, decreases with increasing gate voltage, from ≈ 300 at −9.1 V to ≈60 at −82 V, agreeing qualitatively with the experimental ratio but in a milder way than the experimental data. The discrepancy can be ascribed to that extrinsic effect in experiment such as nonlinear contact resistivity is not taken into account in the simulated device. Nevertheless, the gate-tunable GAR can still be intrinsically analyzed from the calculated transmission coefficient, as shown in Supplementary Fig. 26. At low gate voltage (− 9.1 V), there is almost no *x* direction transmission channels in the scattering region between source energy level *E*_L_ and drain energy level  *E*_R_, whereas a sizeable *y* direction transmission is observed, giving rise to a large anisotropic ratio *Γ*_a_ at low gate voltages. In contrast, at high gate voltage (− 82 V), non-zero transmission coefficient in channel material appears with comparable total transmission in both *x* and *y* directions, greatly suppressing GAR in GaTe.

### Ultra-thin GaTe anisotropic floating gate memory

**Fig. 3:**
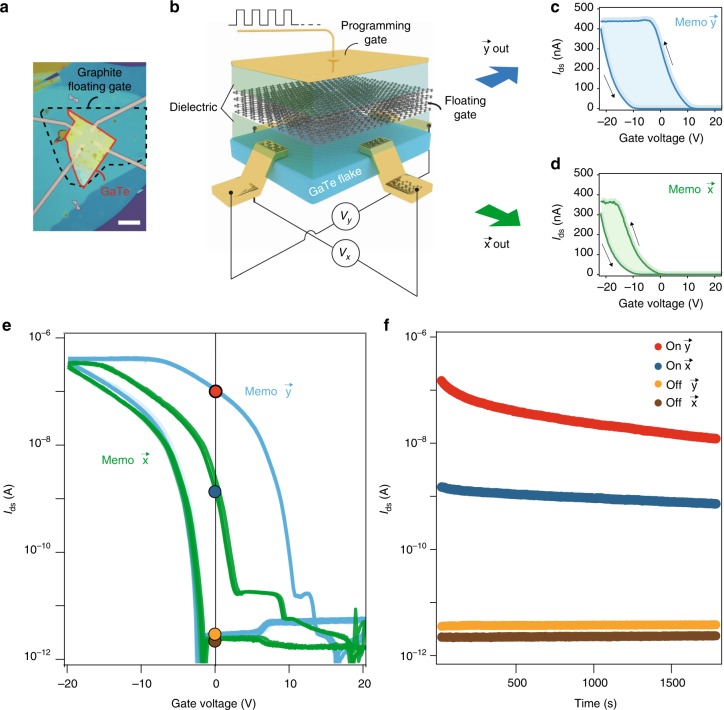
Anisotropic ultra-thin GaTe floating gate memory. **a**, **b** Optical image and art view of a typical h-BN/GaTe/h-BN device (Sample S6) with a graphite floating gate. Scale bar in **a** is 10 μm. **c**, **d** Memory curves measured along *x* and *y* directions, respectively. **e** Same data in **c** and **d**, plotted in log scales, with 10 repeated measurements (indicated by changing the gradient of the green and blue colors). **f** Retention time of memory at *V*_g_ = 0 V, along *y* and *x* directions, with on and off positions indicated by the colored circles in **e**. *V*_ds_ = 2 V was used in the above measurements

It is of great importance to demonstrate conceptual devices taking advantage of the GAR effect in ultra-thin GaTe. In the following, we will discuss a prototype anisotropic memory based on ultra-thin GaTe. Using the vdW assembly method (see Methods), a few-layered graphene was equipped in addition to the h-BN/GaTe/h-BN sandwich, forming an architecture of FGM. Optical image and art view of such typical devices are shown in Fig. 3a, b.

Interestingly, when sweeping within the same gate window, the hysteresis memory curves along *y* and *x* directions vary largely, with a clear discrepancy of memory window size, shown in Fig. 3c, d. This result is more pronounced in a log scale when plotted together in Fig. 3e. One can see that after an operation of erasing (sweep the *V*_g_ from 0 V to −20 V, and back to 0 V), the device stays in an “Off” state in both directions. While an program operation (sweep the *V*_g_ from 0 V to 20 V, and back to 0 V), the device stays in two different “On” states in *y* and *x* directions, as indicated by colored circles in Fig. 3e, and their corresponding retention in Fig. 3f. It thus makes the device an anisotropic memory, i.e., one single operation of programming-erasing will generate two sets of data in the two directions, making the device conceptually compatible with direction-sensitive data storage.

## Discussion

**Fig. 4:**
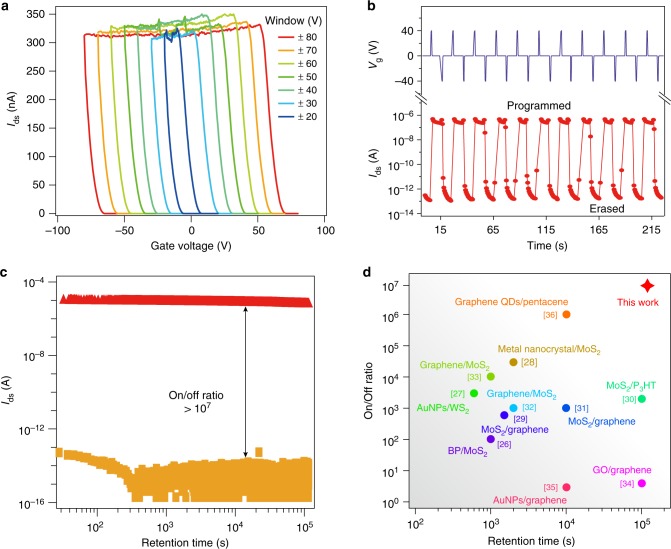
Comparison of the floating gate memory performance of GaTe with other 2D materials. **a** Memory windows measured in *y* direction. **b** Demonstration of erasing and programming pulses measured in *y* direction. **c** Retention time test of the device at on and off states. **d** Summary of the performance of FGM made by 2D materials. Data in **a**, **b** are measured in sample S6, and data in **c**, **d** are measured in sample S4. *V*_ds_ = 2 V was used in the above measurements

To verify the performance of ultra-thin GaTe FGM with graphite floating gate along fixed direction, we recorded polarization gate voltages for different ranges from ±20 V to ±80 V in direction *y*, shown in Fig. 4a (see also Supplementary Figs. 27–28). It can be seen that the GaTe memory shows rather stable erased state in all gate ranges. Once fed with the stimulation of pulsed *V*_g_ of ±40 V, the device can be readily erased and programmed as illustrated in Fig. 4b.

In the following, we compare the GaTe FGM with the state-of-the-art FGMs fabricated from other 2D materials^26–36^. Given that retention of the erased and/or programmed states is one of the most important parameter of such FGM, we recorded along direction *y* the on state (by a positive gate pulse), and the off state (by a negative gate pulse), respectively. Among GaTe FGM devices tested, the best performance (measured from sample S4 as shown in Fig. 4c) exhibits an on/off ratio exceeding 10^7^ and the attenuation of the on/off ratio is < 5% in a retention time of 10^5^ s (detailed memory characterizations of sample S4 are shown in Supplementary Fig. 29). By summarizing the state-of-the-art performance of FGM based on 2D materials, it is found that, our h-BN/GaTe/h-BN with graphite floating gate memory shows up to now the record on/off ratio and retention time, as shown in Fig. 4d. We attribute the observed high on/off ratio in the h-BN encapsulated devices to the better preserved band gap in the pristine GaTe. When the Fermi level is tuned to be inside the band gap (no electron density of state available), the off-state current is at the order of pA, whereas the on-state current reaches a few μA. To illustrate the working principle of the GaTe floating gate memory devices, we show, according to previous studies^26,27,29,33^, simplified band diagram in Supplementary Fig. 30.

In conclusion, we have discovered that ultra-thin GaTe encapsulated by h-BN devices show a twofold symmetry electrical conductance oscillations along different in-plane directions, with their maximum anisotropic ratios *I*_y_/*I*_x_ gate-tunable in the range from < 10 to a few thousands. This GAR arises from the in-plane anisotropic transmission coefficients, and the anisotropic ratio can be changed by several orders of magnitude mainly owing to the sensitivity of *x* direction conduction channel to gating. Based on this GAR effect, we devised an anisotropic GaTe memory with a vdW assembled graphite floating gate. The prototype memory devices show direction-sensitive multiple-level output non-volatile memory behavior when measured from different directions. Moreover, fixed direction measurements suggest that the GaTe FGMs show record performances in terms of both on/off ratio and retention time among the state-of-the-art FGM based on 2D materials. Using this unit cell of GaTe anisotropic field effect transistor as a building block, it can be further expanded into many possible applications such as neural computation with GaTe FGM neural network arrays.

## Methods

Single crystal GaTe was prepared via the self-flux method. Raw bulk materials with stoichiometric ratio of Ga (purity 99.999%): Te (purity 99.9%) = 48.67:51.33 were mixed and kept at 880 °C for 5 h. The mixture was then cooled at the rate of 1.5 °C h^−1^, followed by a natural cooling process. The h-BN (crystals from HQ Graphene) encapsulated GaTe devices were fabricated using the dry-transfer methods (Supplementary Fig. 31)^37^, and then annealed in Ar:H_2_ (10:1) mixture at 320 °C for 30 mins before electrical measurements.

The h-BN-encapsulated GaTe heterostructures were fabricated in an mBraun-universe glove box. A Bruker Dimension Icon AFM was used for thicknesses and morphology tests. Raman measurements were performed by an HR 800 JobinYvon Horiba polarized Raman spectroscopy. The electrical performances of the devices were measured using a semiconductor analyzer (Agilent B1500A) and a probe station (Cascade Microtech Inc. EPS150) under ambient conditions.

The electronic band structure in this work was calculated by using the first-principles density functional theory as implemented in the VASP code^38^. Projector augmented wave pseudopotentials^39^ and the Perdew-Burke-Ernzerhof^40^ functional were, respectively, used to describe electron–ion interaction and electronic exchange-correlation interaction. We adopted 500 eV as the electronic kinetic energy cutoff for the plane-wave basis and 10^−6^ eV as the criterion for reaching self-consistency. The Brillouin zone (BZ) of the primitive unit cell (12 atoms per cell) was sampled by 20 × 20 × 1 k-points^41^. Rectangle supercell (24 atoms per cell) was used to calculate anisotropic electrical conductivity and the BZ was sampled by 2 × 6 × 1 k-points.

The electrical conductivity *σ* was evaluated based on *σ* = ne*μ*, in which carrier mobility *μ* was calculated based upon the deformation potential theory with formula^23,24^

1 $$\mu = \frac{{e\hbar ^3C_{2D}}}{{k_BTm^ \ast m_dE_1^2}},$$

where ℏ is the reduced Planck constant, *C*_2D_ is the elastic modulus derived from (*E* − *E*_0_)/*A*_0_ = *Cε*^2^/2 (*E*, *E*_0_, *A*_0_, *C*, and *ε* denote, respectively, the total energy, the total energy at equilibrium, the area of the 2D unit cell at equilibrium, elastic constant, and the lattice strain), *k*_B_ is the Boltzmann constant, *T* is temperature, m^*^ is the effective mass in the transport direction, *m*_d_ is the averaged effective mass defined by $m_d = \sqrt {m_x^ \ast m_y^ \ast }$ and *E*_1_ is the deformation potential constant of the VBM along the transport direction. *E*_1_ defined by *E*_1_ = Δ*V/ε* with Δ*V* as the energy change of the VBM under small strain *ε*.

*I–V* curve of the GaTe transistor was simulated using the DFT coupled with the NEGF method^25^, as implemented in the ATK package^42^. The structure of GaTe transistor is shown in Supplementary Fig. 25(a). We employed the Dirichlet boundary condition to ensure the charge neutrality in the source and the drain region. The temperature was set to 300 K. The density mesh cutoff was set to 80 Hartrees. The Monkhorst-Pack k-point meshes for device along the *x* and *y* direction were sampled with 40 × 6 × 1 and 114 × 6 × 1, respectively. Electron–ion interaction was treated by SG15 Optimized Norm-Conserving Vanderbilt (ONCV) pseudopotentials^43,44^. The transmission coefficient at energy E was averaged over 31 k-points perpendicular to the transport direction. The generalized gradient approximation was adopted for the electronic exchange-correction functional. The length of scattering region is 9.530 nm and 9.676 nm for transistor along *x* and *y* directions, respectively, both of which are sufficiently large to avoid interaction between source and drain. The code of VASP (www.vasp.at) and QuantumATK (https://docs.quantumwise.com/guides/guides.html) are commercially available from their official websites.

## Supplementary information

Supplementary Information

## Data Availability

The data that support the findings of this study are available from the corresponding authors upon reasonable request.
