# Supplementary material for: Gate tunable giant anisotropic resistance in ultra-thin GaTe
Source: Nat Commun. 2019 May 24;10:2302. doi: 10.1038/s41467-019-10256-3 (PMC6534542; doi:10.1038/s41467-019-10256-3)
Supplement: Supplementary file 1 — Supplementary Information [file 41467_2019_10256_MOESM1_ESM.pdf]

Supplementary Information for

"Gate tunable giant anisotropic resistance in ultra-thin GaTe"

Hanwen Wang, *et al*

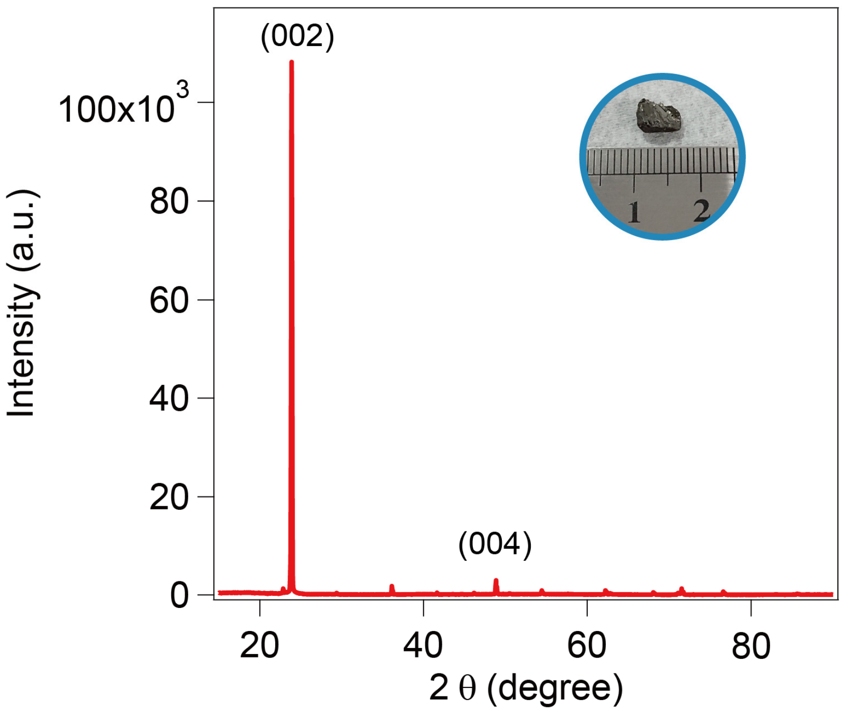

Supplementary Figure 1. X-ray diffraction (XRD) pattern of GaTe single crystal (the optical image of the crystal is shown in the inset).

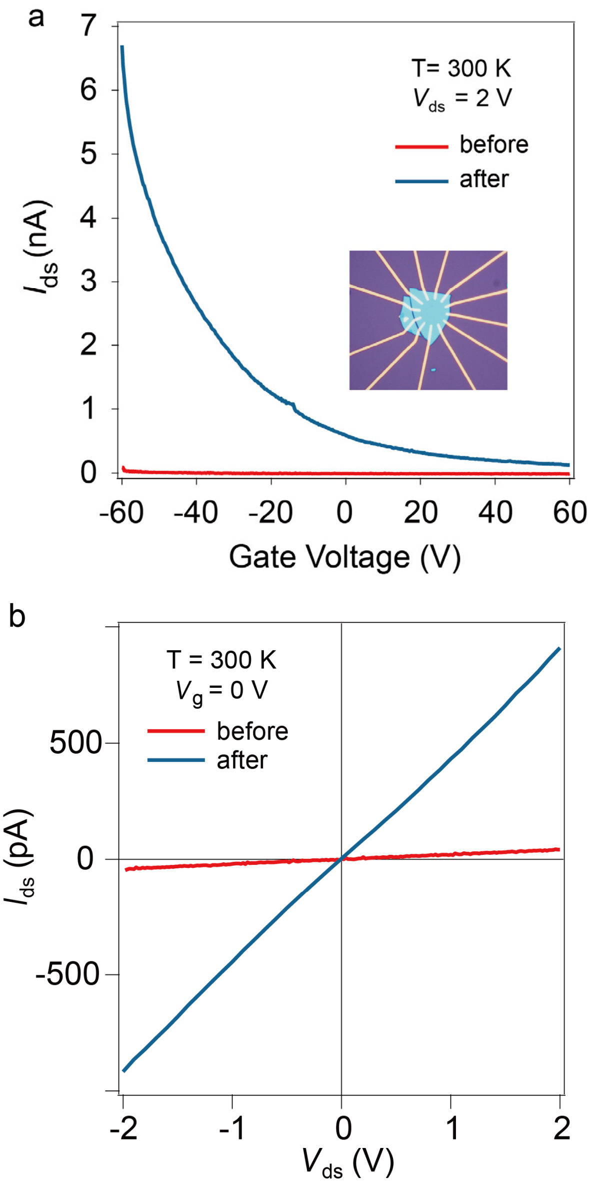

Supplementary Figure 2. Annealing effects on the electrical properties of the GaTe device. (a) The field effect curves of the device before and after annealing. (b) The *I-V* characteristics of the device before and after annealing. It was found that by thermal annealing at 320 ^{o}C in forming gas (Ar:H_2=10:1) for 30 mins, the conductivity is much enhanced in the GaTe ultra-thin flakes. Optical image of the measured device is given in the inset of Fig. S2(a).

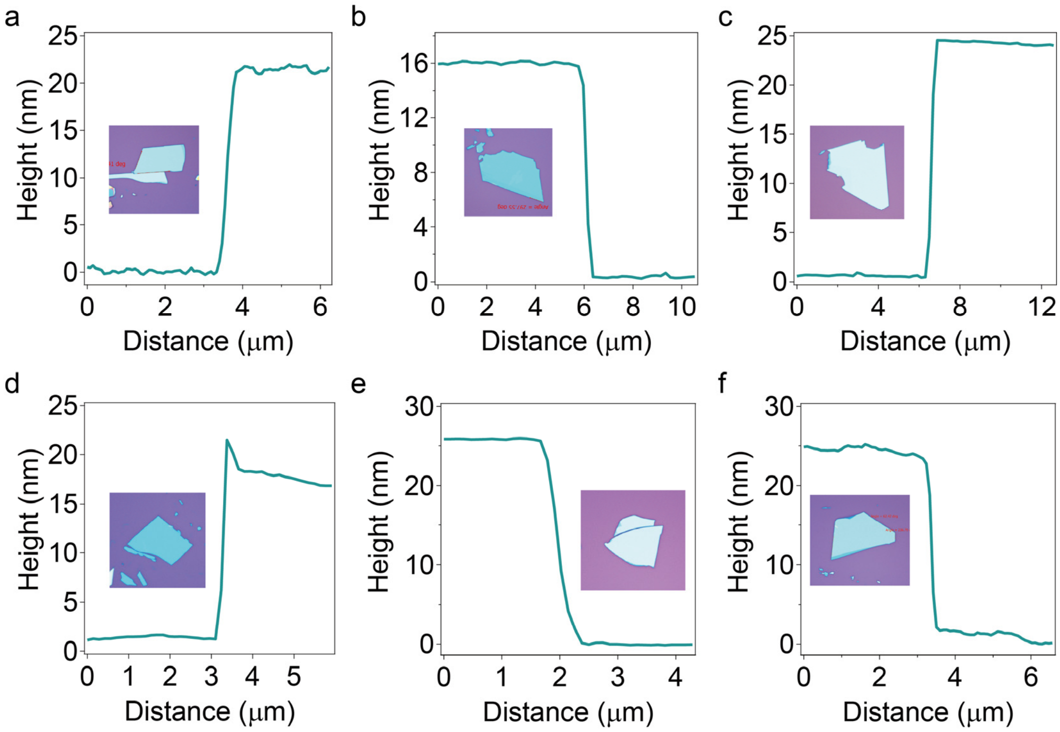

Supplementary Figure 3. (a)-(f) Optical images and Atomic-force microscope line scans (height profiles) of typical GaTe flakes among devices that we studied in this work.

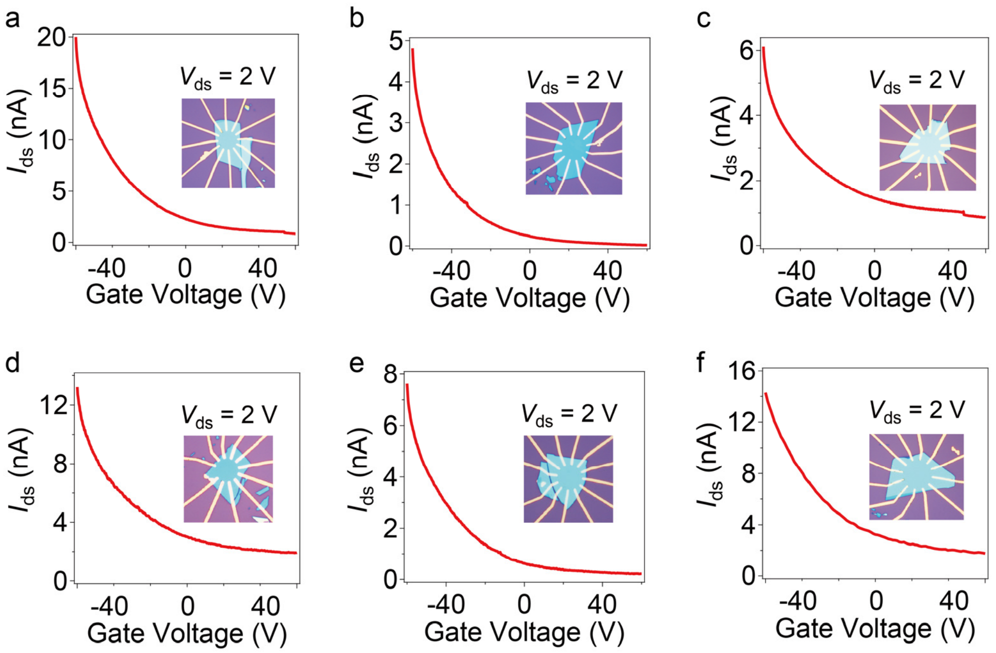

Supplementary Figure 4. (a)-(f) Field effect curves of the 6 devices listed in Supplementary Figure 3. Inset is a typical optical image of such devices. Measurements are carried out at room temperature. Devices are fabricated in air without h-BN encapsulations. Devices are annealed using the same parameters described in Supplementary Figure 2.

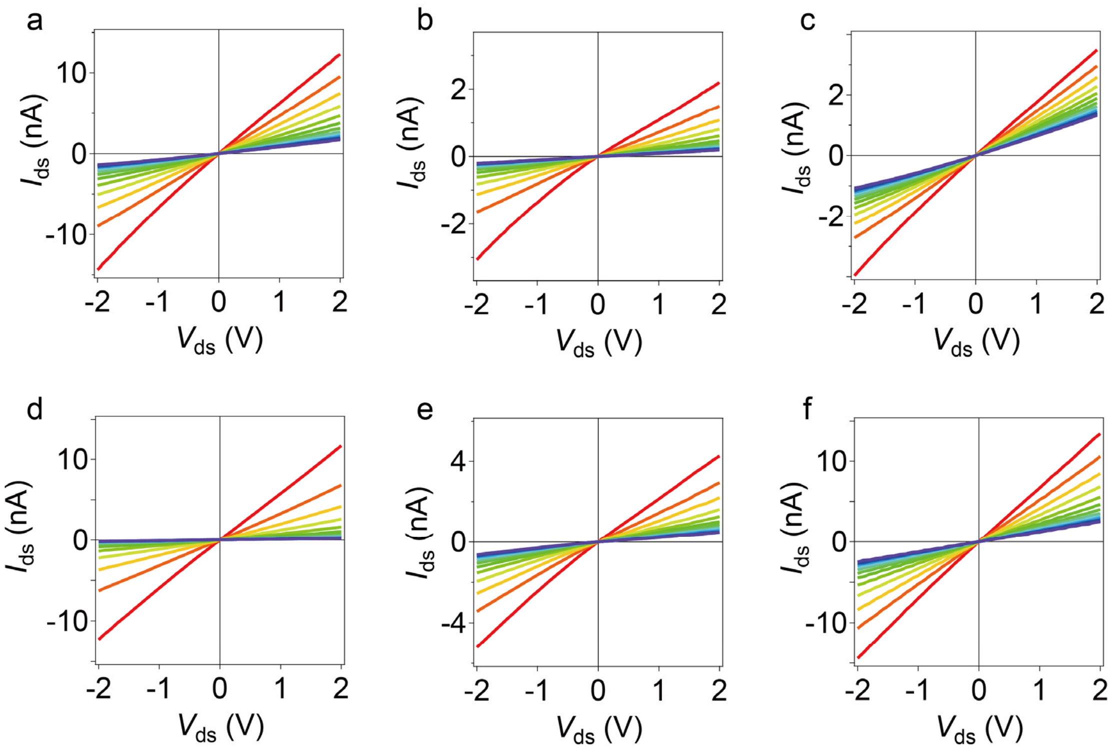

Supplementary Figure 5. (a)-(f) *IV* characteristics at different gate voltages of the corresponding devices in Supplementary Figure 4.

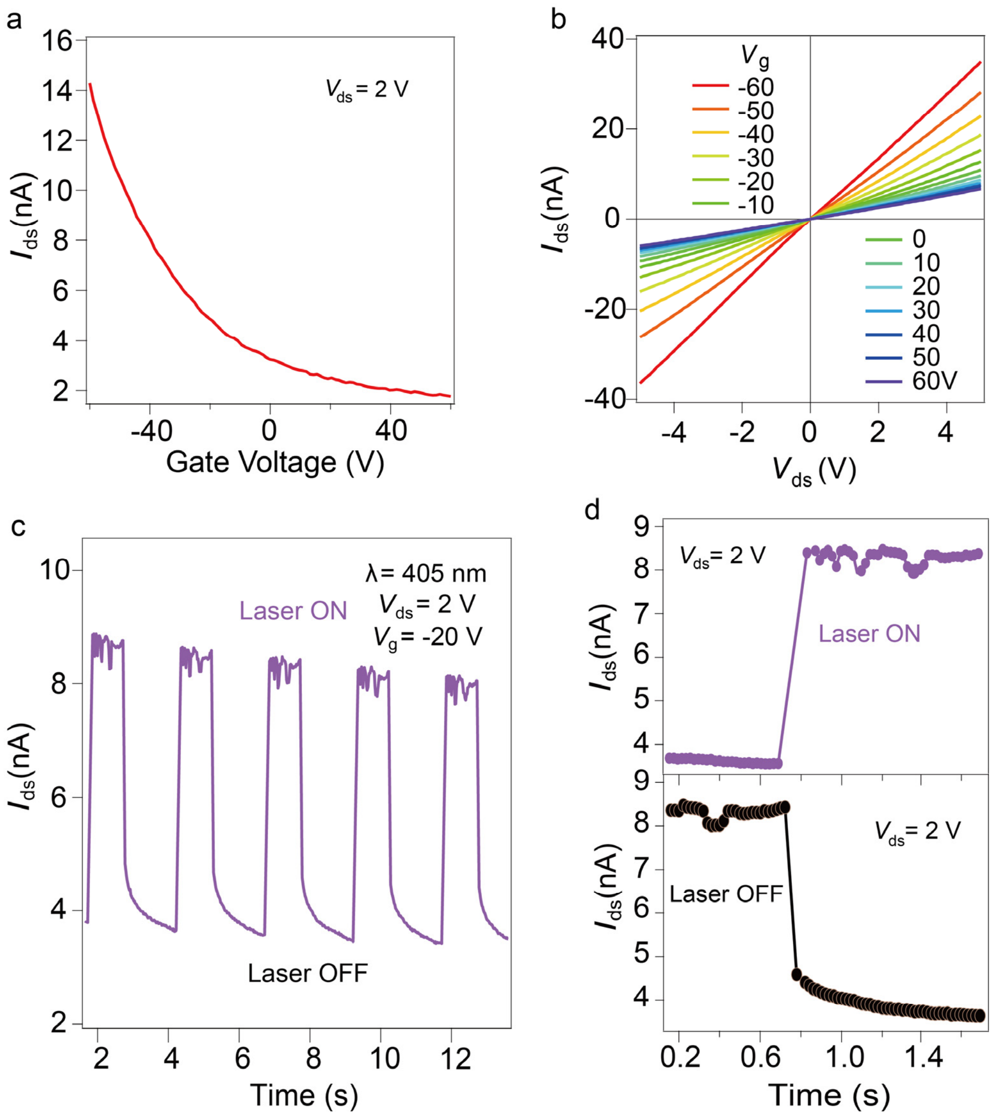

Supplementary Figure 6. The photoresponse of a typical GaTe device without BN encapsulation. (a,b) The transfer and output curves of the device. (c) Time resolved photoresponse of the device at V_g=-20V. (d) Typical rise and decay behaviors of the photocurrent with intervals of 20 ms.

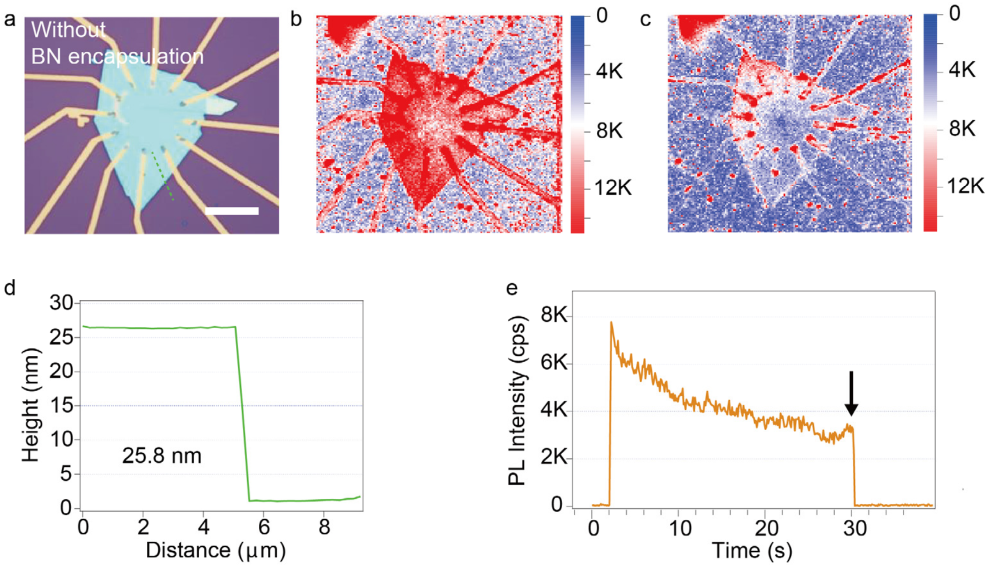

Supplementary Figure 7. Photoluminescence (PL) of ultra-thin GaTe flake exposure to air. (a) Optical image of a typical device (sample S11) made of 25.8 nm GaTe flake, with its height profile plotted in (d). (b)-(c) The first and second time confocal PL maps of the device at room temperature (laser power P=0.05 mW and laser wavelength λ=532 nm). Scale bar in (a) is 10 μm. (e) PL of the GaTe flake recorded in a time-series, with the laser focused on the GaTe area. The arrow indicates the switch-off of the laser. It is seen that PL intensity of GaTe flake exposed to air (with the thickness of 25.8 nm) is rather weak. Worse still, the PL signal will be quickly decaying, even shortly after a confocal scanning process. As reported in the previous work [1], the weak PL signal and the fast PL decay can be attributed to the formation of oxide-decomposition products (such as oxygen chemisorbs to tellurium).

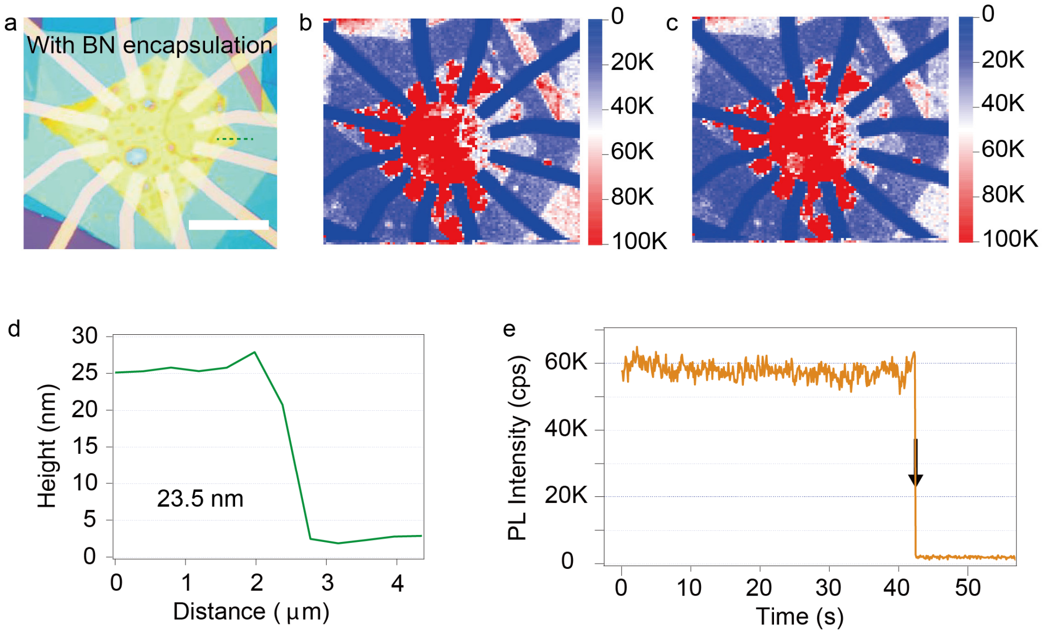

Supplementary Figure 8. PL characterizations of ultra-thin GaTe flake encapsulated by h-BN. (a) Optical image of a typical device made of 23.5 nm GaTe flake, with its AFM height profile plotted in (d). (b)-(c) The 1st and 10th confocal PL maps of the device at room temperature (laser power P=0.05 mW and laser wavelength λ=532 nm). Scale bar in (a) is 10 μm. (e) PL of the GaTe flake recorded in a time-series, with the laser focused at a fixed spot on the GaTe area. The arrow indicates the switch-off of the laser. Compared to Supplementary Figure 7, it is seen that for the GaTe flake encapsulated by h-BN, its PL intensity is about one order of magnitude stronger than that of the samples exposed to air. The stability of PL signal for the GaTe flake encapsulated by h-BN can be determined by the multiple confocal PL maps and the monitor of PL time-series. Thus, we believe that no obvious oxidation occurred to the GaTe flakes encapsulated by h-BN in a glove box.

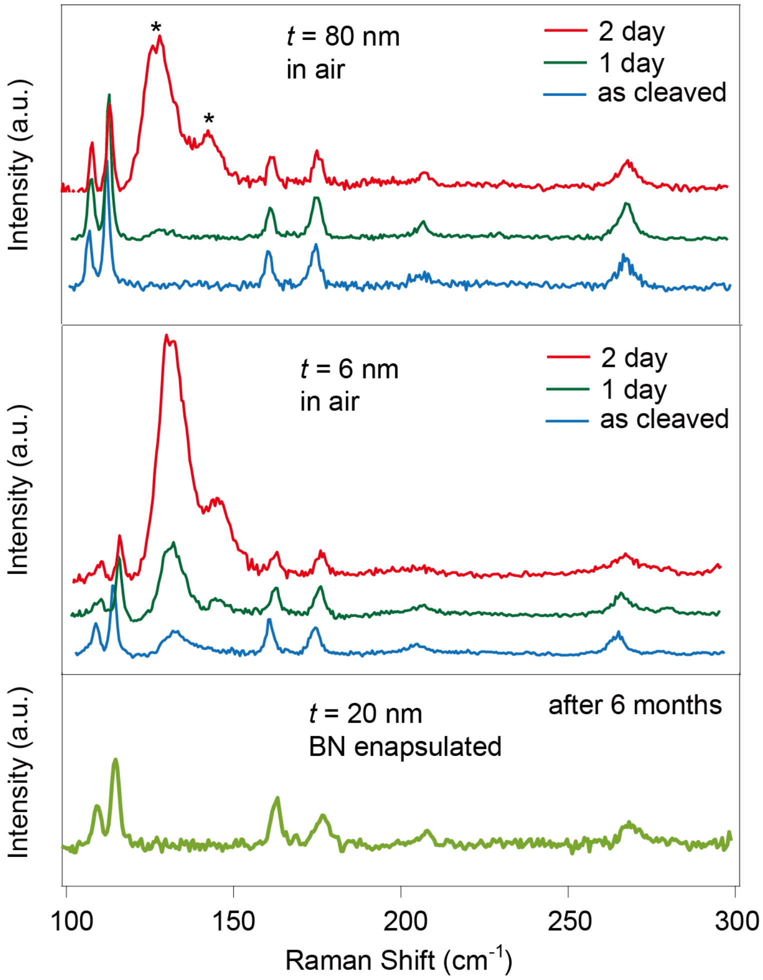

Supplementary Figure 9. Raman spectra of GaTe flakes with different conditions. Upper and middle panels correspond to GaTe flakes exposed to air, with thicknesses of 80 and 6 nm, respectively. Bottom panel shows the Raman spectrum of 20 nm GaTe encapsulated in h-BN and measured after 6 months. It is well established that in thin GaTe flakes, Raman peaks evolve rapidly with respect to time exposed to air, with two broad peaks (indicated by stars in the upper panel) develops at ~131 and ~145 cm^{-1} [Ref.1]. For GaTe flakes exposed to air the degradation peaks develops rapidly in the time scale of less than a few days. However, for the devices fabricated in glove box and protected by h-BN encapsulation, the GaTe flake can retain their pristine Raman Spectra for months, without any extra peaks correlated to degradation.

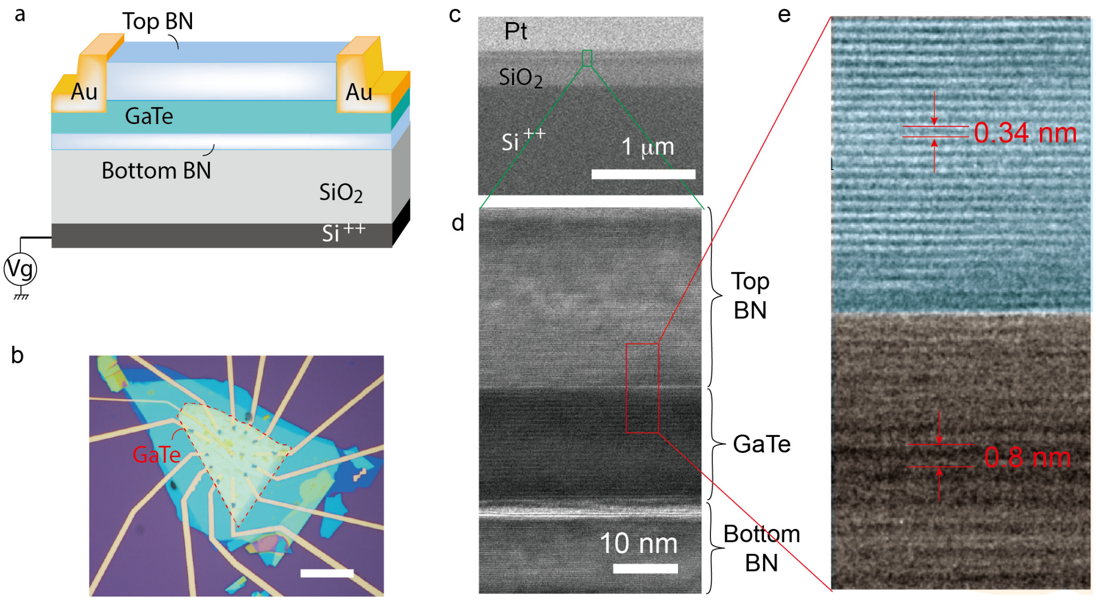

Supplementary Figure 10. Transmission Electron Microscopy (TEM) cross-section of ultra-thin GaTe flake encapsulated by h-BN. (a) Cartoon image of a typical device. (b) Optical image of the h-BN/GaTe/h-BN device, which is used for TEM cross-section observation in (c)-(e). It is seen that in the TEM cross-section, a sandwich structured h-BN encapsulated GaTe can be clearly observed. Thanks to the BN protection, the GaTe part (device stored for months) displays an inter-layer space of 0.8 nm, which is in agreement of its pristine bulk lattice parameters [2]. TEM lamellas were prepared on an FEI Helios NanoLab 400S FIB/SEM dual-beam system equipped with a Ga+ ion source. Pt layers were deposited on the surface region of interest by Electron&Ion beam for sample protection.

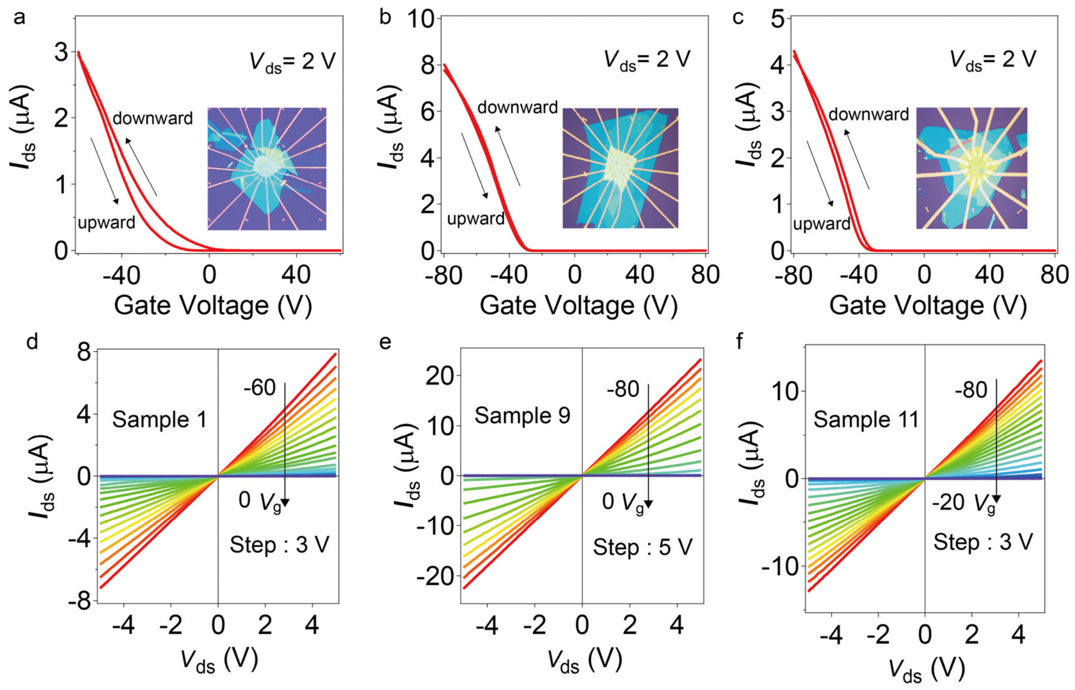

Supplementary Figure 11. Electrical properties of three typical devices made by GaTe flakes encapsulated by h-BN. It can be seen that electrical conductivity is significantly improved compared to those fabricated without h-BN encapsulation, as shown in Supplementary Figures S2-6. (a)-(c) Field effect curves of the devices with their optical images shown in each inset. (d)-(f) *IV* curves at different gate voltages of the corresponding devices in (a)-(c).

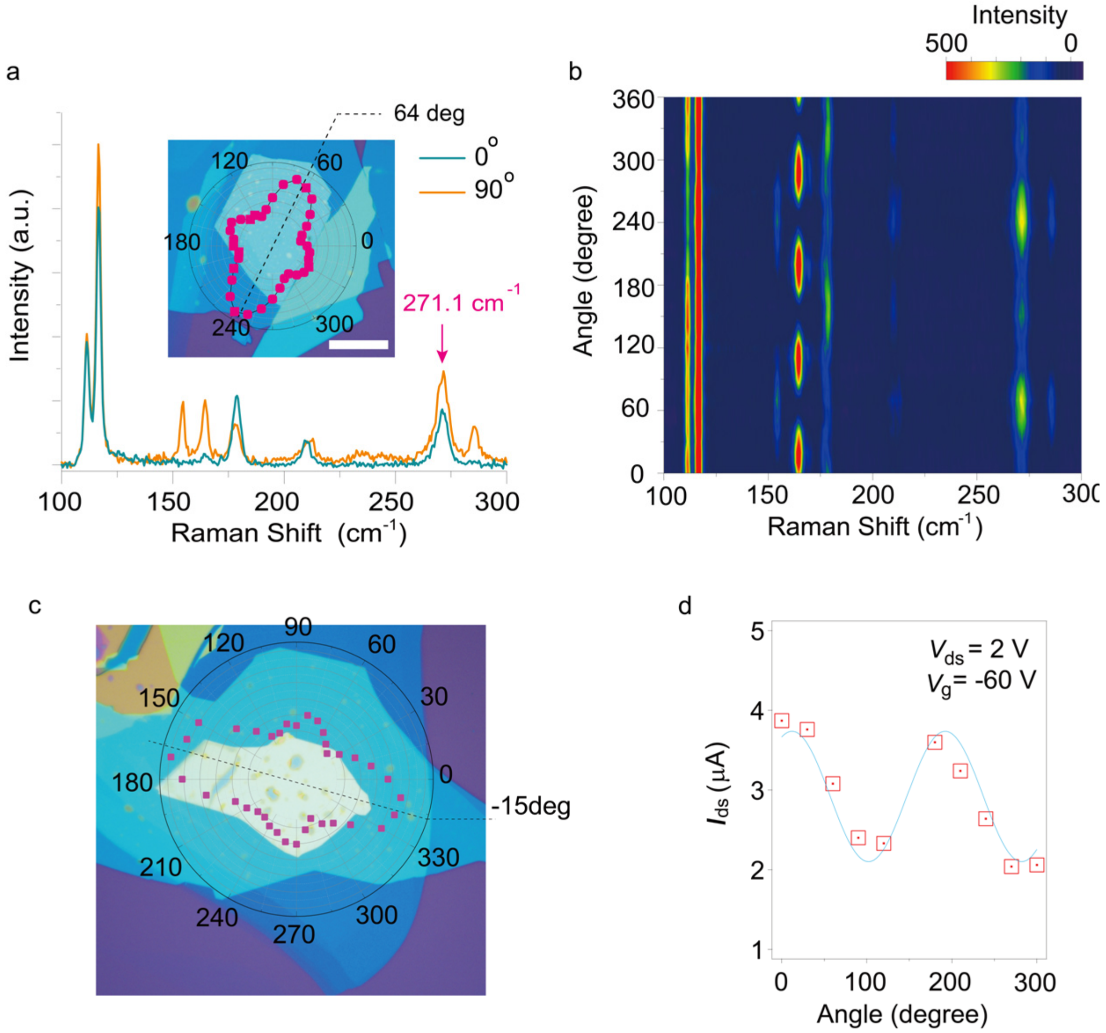

Supplementary Figure 12. (a) Polarized Raman measurement of a typical GaTe flake encapsulated by h-BN (sample-S1 in the main text). Inset shows the Raman intensity at Raman shift of 271.1cm^{-1}, plotted in a polar graph. (b) Color map of the polarized Raman profile as a function of rotation angle. The 64 deg angle in the polarized Raman spectroscopy is parallel to the exfoliated straight edge of the GaTe flake. This straight edge is found to be the direction which allows a maximum conductivity in further electrical measurements in Fig.1 in the main text. We define this direction as ***y***-axis, according to the following analysis in Supplementary Figure 13. (c) Optical image and the Raman intensity at 271.1 cm^{-1} for sample-S3. (d) I_{ds} as a function of angle at V_g=-20 V for the device made from the h-BN/GaTe/h-BN stack in (c). The optical image of the final device of sample-S3 is shown in Supplementary Figure 18a.

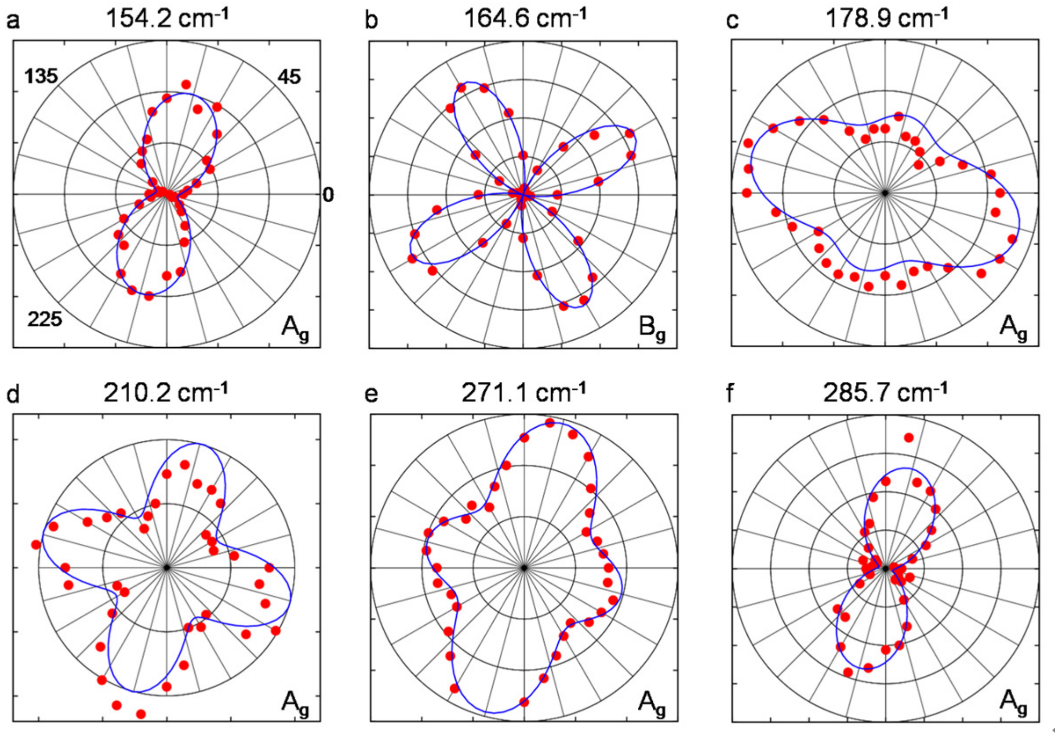

Supplementary Figure 13. The Raman intensity *v.s.* polarization angle θ for six typical types of Raman modes. Experimental data and the corresponding theoretical fittings are shown in red solid dots and blue lines, respectively. Polarization dependent Raman intensity of A_g modes in (a), (c), (d)-(f), and B_g modes in (b) usually take the form of $[a*cos^2(\theta-\phi) + b*sin^2(\theta-\phi)]^2$ *and* $[e*sin(2(\theta-\phi))]^2$, respectively. Clear anisotropic Raman modes can be seen, especially from A_g mode, whose maximal intensity gives the principal axis of crystal. For example, from A_g mode at (a), (e), (f), the **y**-axis (crystalline structure indicated in Fig. 1 in the main text) of the studied GaTe flakes can be unanimously assigned. Also, by comparing with Ref. [3], we can conclude that, from the results shown in Supplementary Figure 11-12, the **y**-axis is along the direction of maximum conductivity.

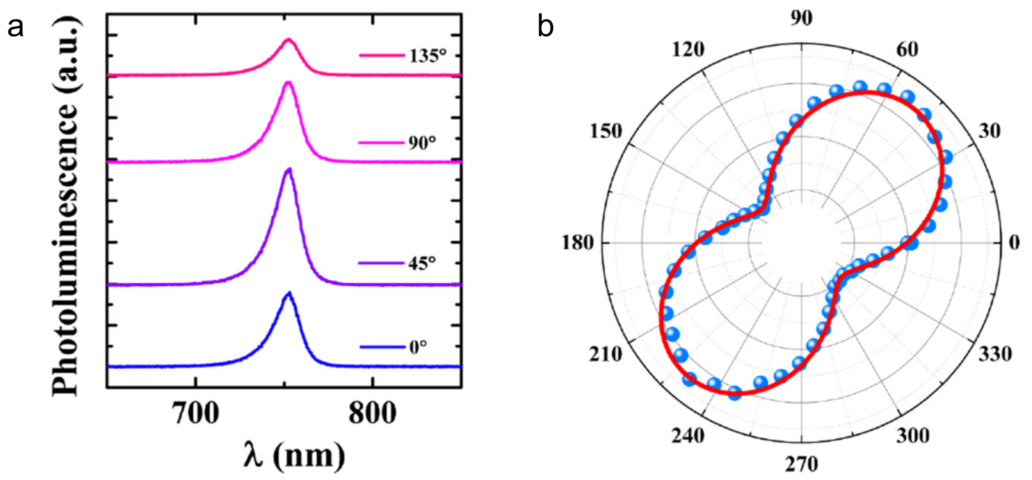

Supplementary Figure 14. Anisotropic photoluminescence of GaTe. (a) Excitation polarization dependence of the PL spectra for θ of 0°, 45°, 90°, and 135°. (b) Polar plots of the PL intensity as a function of θ. Owing to the reduced in-plane symmetry, the photoluminescence (PL) intensity of layered GaTe also exhibits strong polarization dependence. (a) The spectra of a ~ 26-nm-thick GaTe flake, which was obtained by varying the polarization angle of the excitation laser θ and detecting the whole PL signal. There are no obvious shifts in the peak positions when tuning the angle. However, the peak intensity of PL varies significantly, with a period of 180°. This dependence can be clearly observed in the polar plots of the peak intensity ad a function of the polarization angle, as shown in (b). The experimental data can be fitted by a $\cos^2\theta$ function [4], as indicated by the solid line in the plot. The polarization-dependent characteristics of the PL is consistent with previous investigations of layered GeS and $ReSe_2$ [5,6]. (This phenomenon mainly comes from the emission process due to the anisotropic band structure along the zigzag and armchair directions of GaTe [5].)

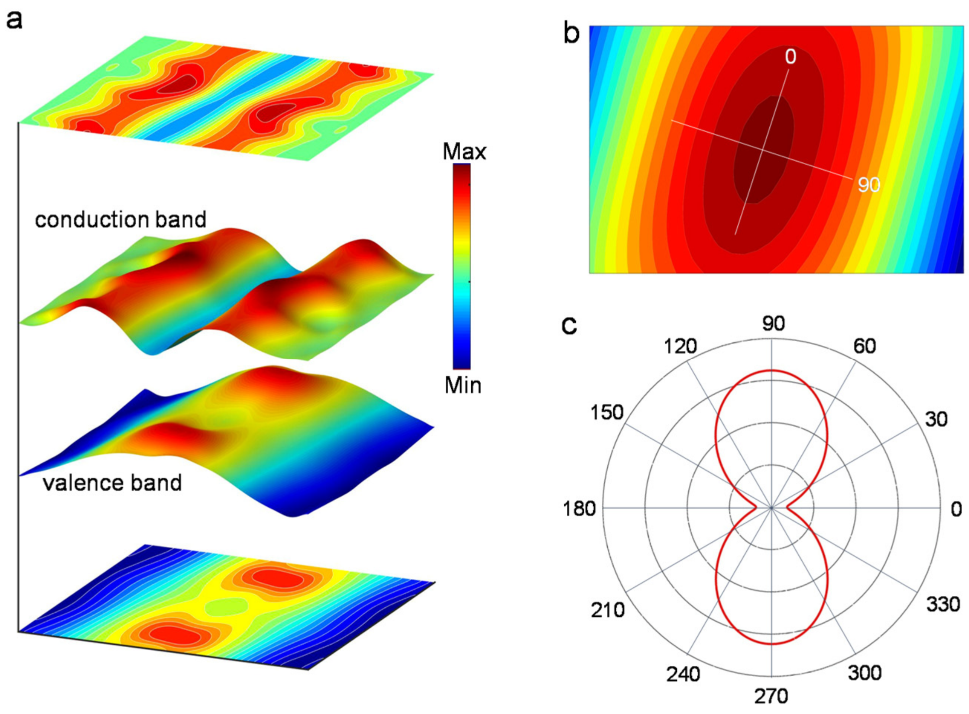

Supplementary Figure 15. (a) The 3D electronic top valence band and bottom conduction band for single layer GaTe. The two outmost panels show the projected contour plot of the two bands, with upper and lower panels for the conduction and valence band, respectively. (b) The contour plot of valence band near the valence band maximum (VBM). (c) The polar plot of $1/m^*$ of carrier at VBM.

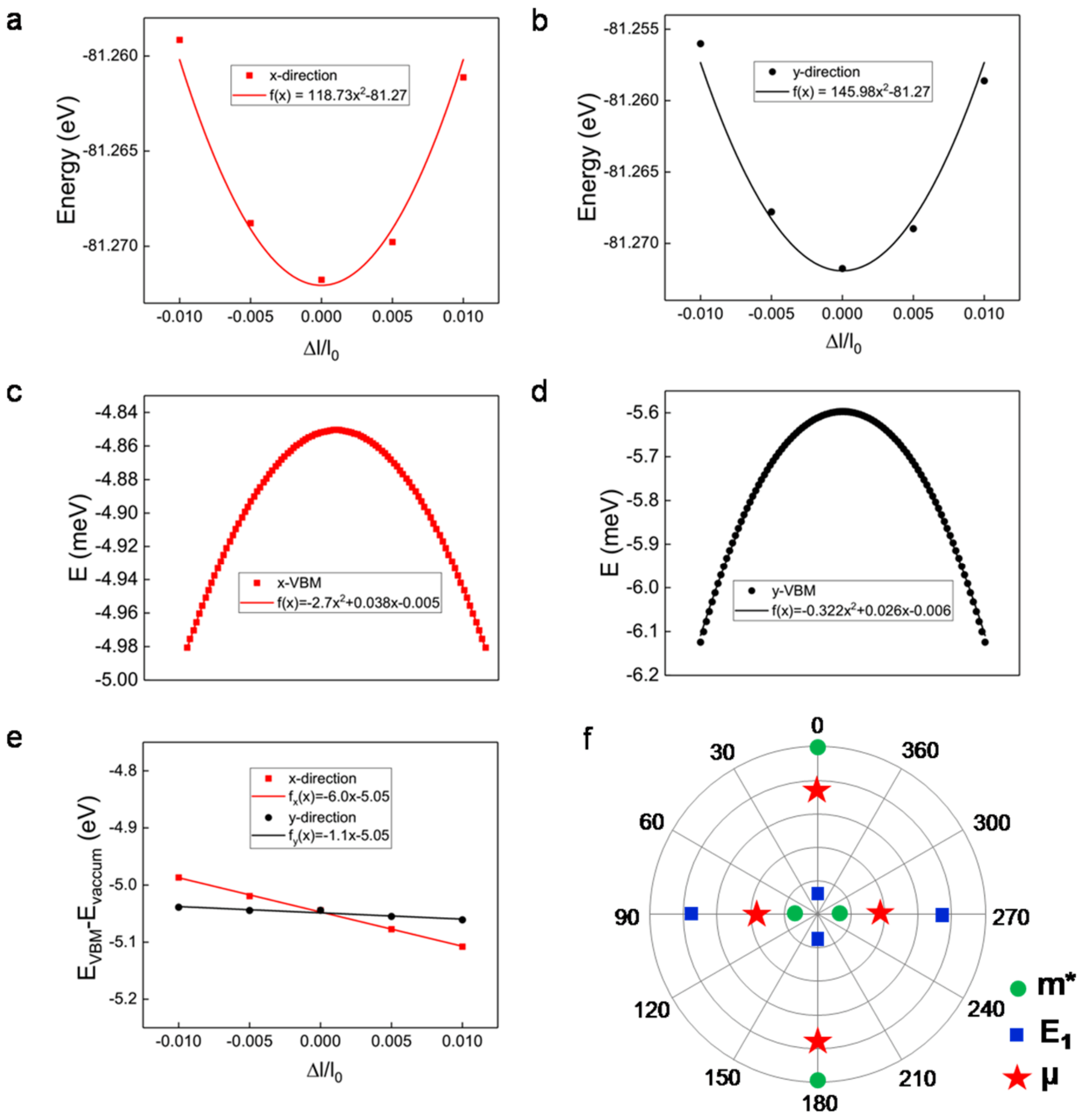

Supplementary Figure 16. The anisotropic parameters for carrier mobility calculation. (a,b) The total energy as a function of deformation l/l_0 to extract elastic modulus along $\boldsymbol{x}$ and $\boldsymbol{y}$ directions, respectively. (c) and (d) The energy dispersion near VBM along $\boldsymbol{x}$ and $\boldsymbol{y}$ direction are used to calculate effective mass m^*. (e) Anisotropic deformation potential E_1 along $\boldsymbol{x}$ and $\boldsymbol{y}$ directions. (f) Anisotropic m^*, E_1, and mobility μ along two perpendicular directions in polar plot.

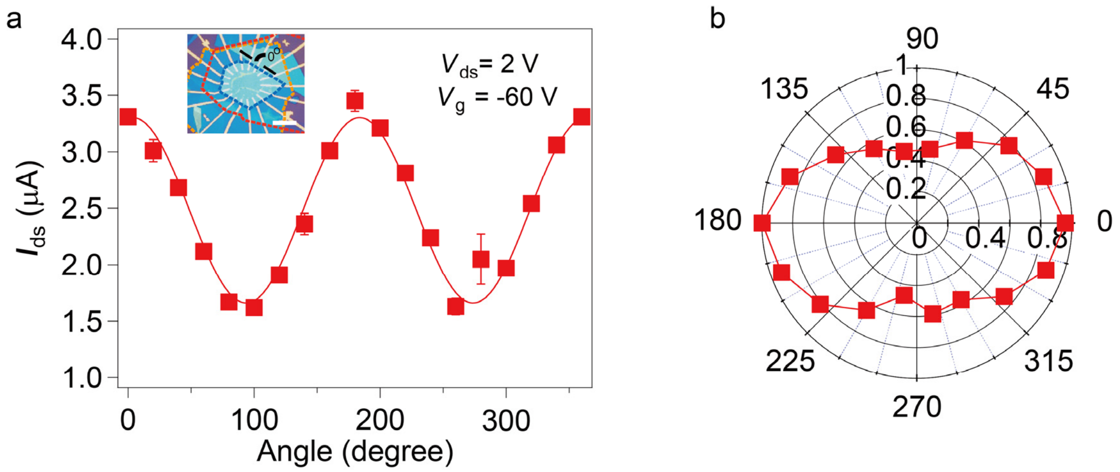

Supplementary Figure 17. Electrical anisotropy of the sample 1 before patterning (same as the status in Fig. 1e in the main text). (a) Source drain current as a function of angle at V_g=-60V. The red solid line is fitted by $\sigma_\theta = \sigma_x \cos^2(\theta-\phi) + \sigma_y \sin^2(\theta-\phi)$ [7]. Inset shows the optical image of the device. (b) Same data in (a) plotted after re-normalization in a polar graph.

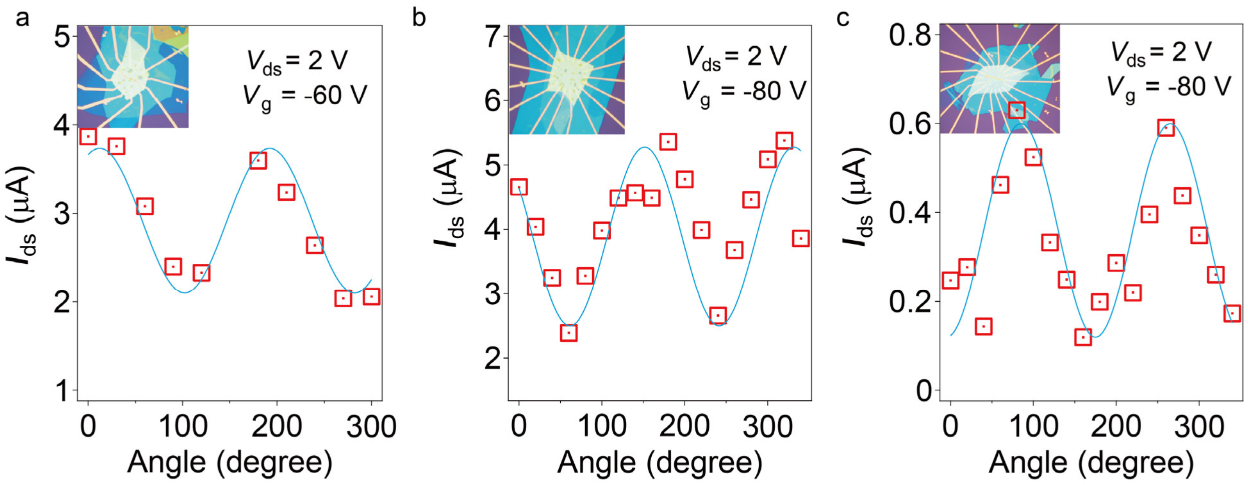

Supplementary Figure 18. (a)-(c) Electrical anisotropy of three typical devices made by ultra-thin GaTe flakes encapsulated by h-BN, with their corresponding optical images shown in each inset.

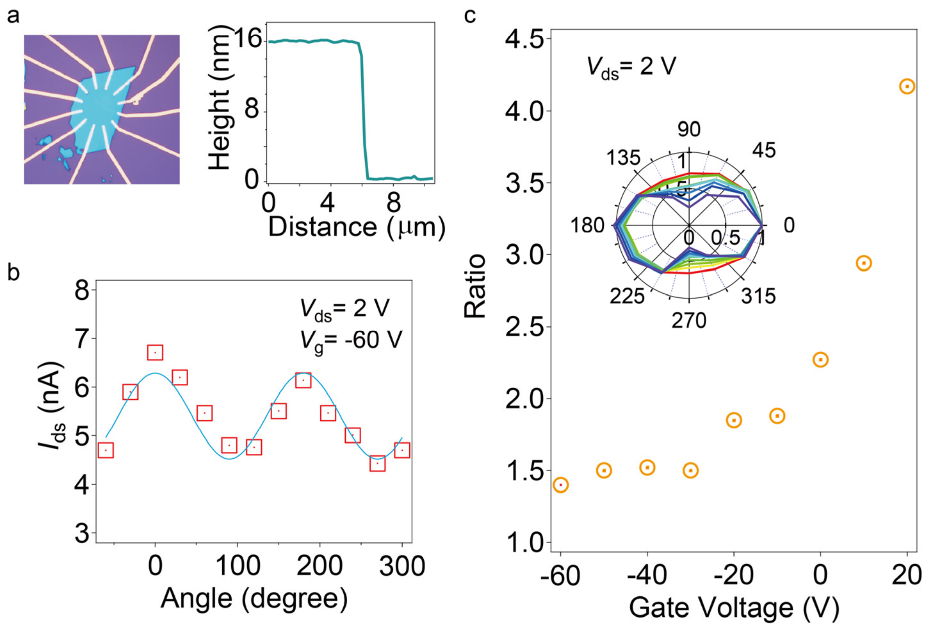

Supplementary Figure 19. Gate-tunable anisotropic resistance in a bare GaTe device without h-BN encapsulation. (a) Optical image and Atomic-force microscope line scans (height profiles) of the GaTe device. (b) Source drain current as a function of angle at V_g = -60 V. (c) The electrical maximum anisotropic ratio I_y/I_x as a function of gate voltage. Inset shows the re-normalized source drain current as a function of angle at different gate voltages plotted in a polar graph.

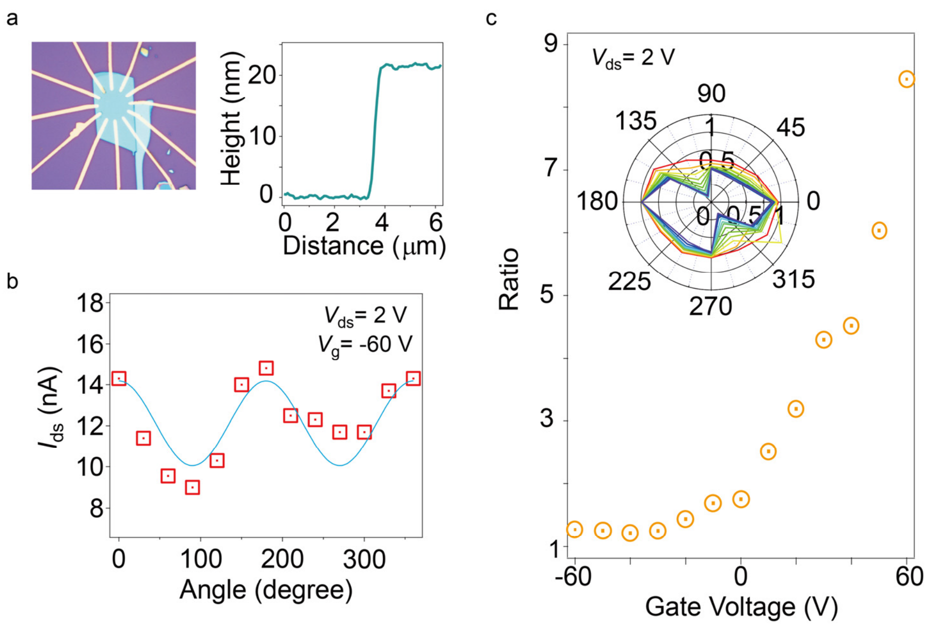

Supplementary Figure 20. Gate-tunable anisotropic resistance in another bare GaTe device. Data presented in the same way as shown in Supplementary Figure 19. (a) Optical image and Atomic-force microscope line scans (height profiles) of the GaTe device. (b) Source drain current as a function of angle at V_g = -60 V. (c) The electrical maximum anisotropic ratio I_y/I_x as a function of gate voltage. Inset shows the re-normalized source drain current as a function of angle at different gate voltages plotted in a polar graph.

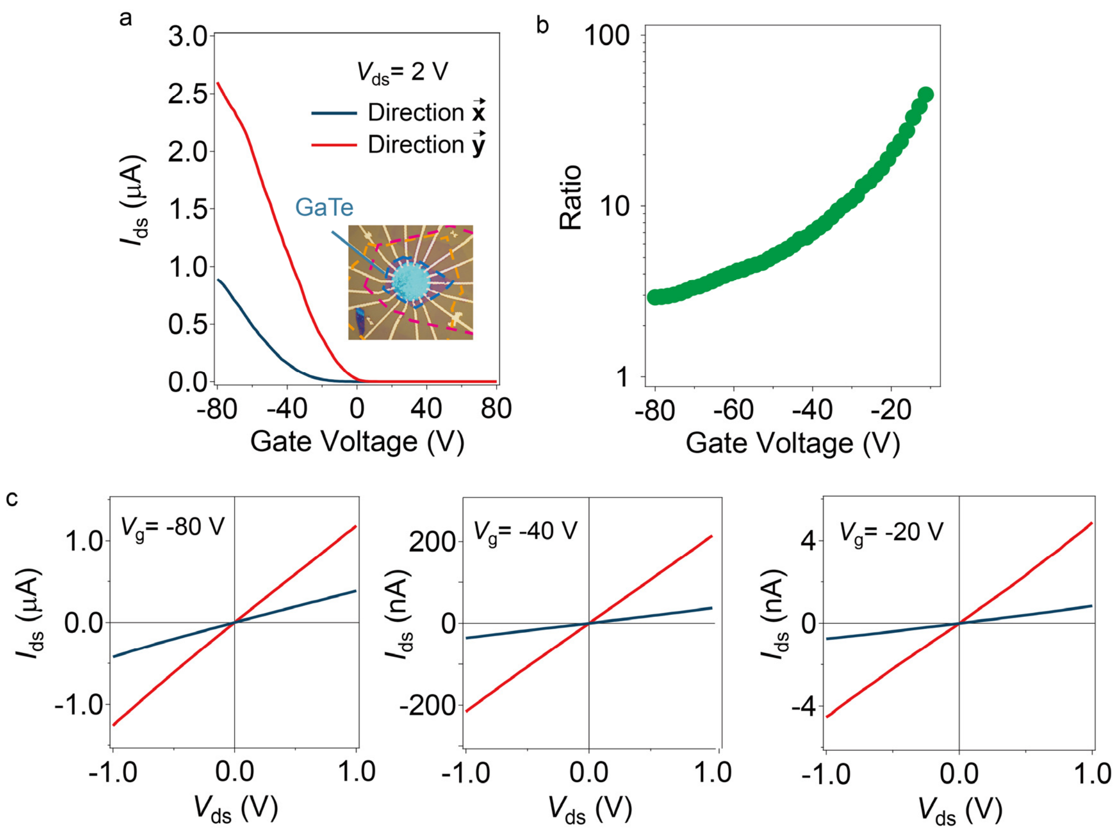

Supplementary Figure 21. Gate tunable giant anisotropic resistance in ultra-thin GaTe encapsulated by h-BN (Sample-S2). (a) Field effect curves of the GaTe device recorded along **x** and **y** directions, with the optical image of the device shown in the inset. (b) The electrical maximum anisotropic ratio I_y/I_x as a function of gate voltage. (c) *IV* curves of the same device measured along **x** and **y** directions at different gate voltages.

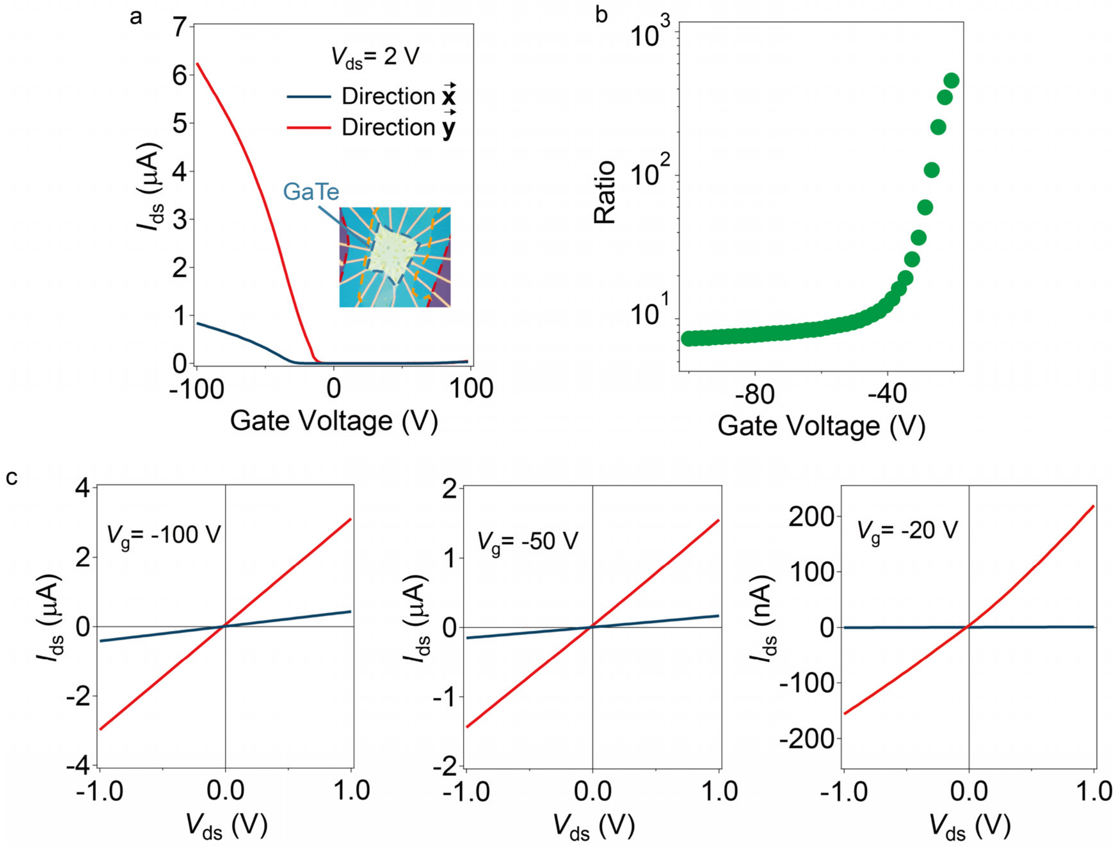

Supplementary Figure 22. Giant anisotropic resistance in ultra-thin GaTe encapsulated by h-BN (Sample-S9). (a) Field effect curves of the GaTe device recorded along **x** and **y** directions, with the optical image of the device shown in the inset. (b) The electrical maximum anisotropic ratio I_y/I_x as a function of gate voltage. (c) *IV* curves of the same device measured along **x** and **y** directions at different gate voltages.

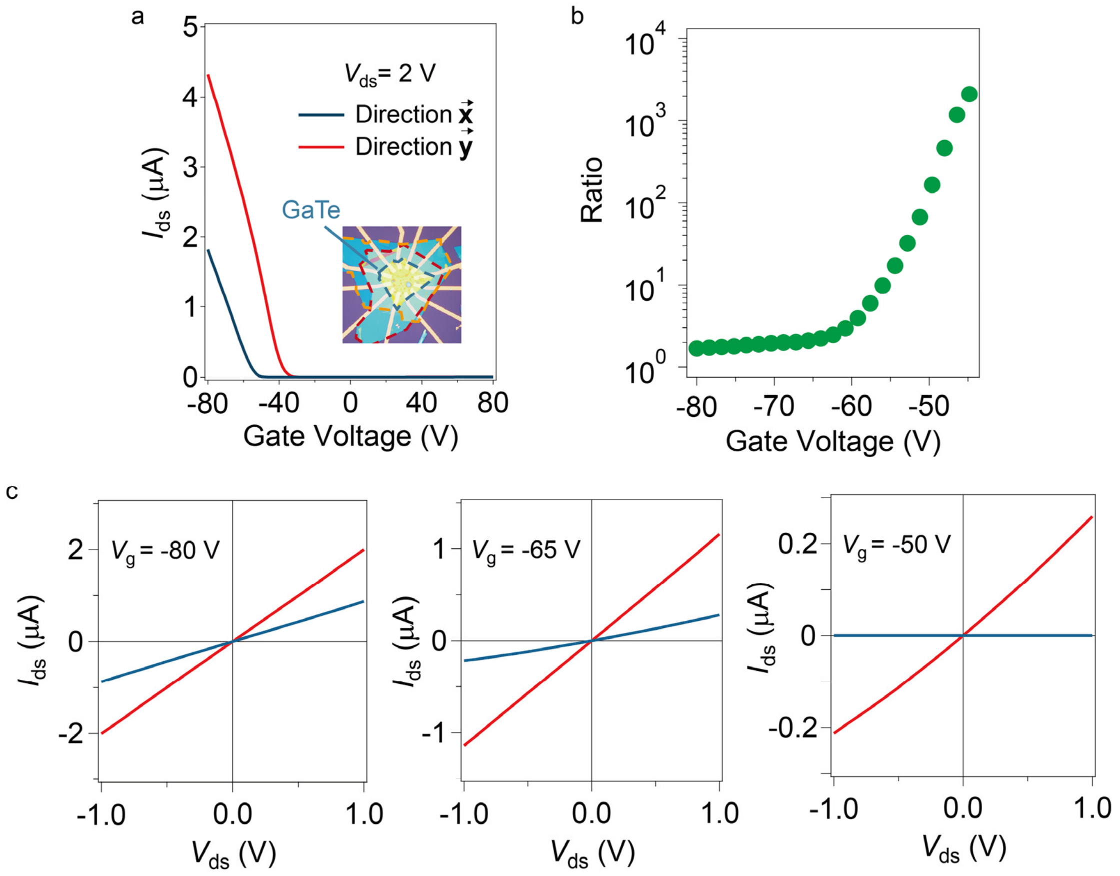

Supplementary Figure 23. Giant anisotropic resistance in ultra-thin GaTe encapsulated by h-BN (Sample-S11). (a) Field effect curves of the GaTe device recorded along **x** and **y** directions, with the optical image of the device shown in the inset. (b) The electrical maximum anisotropic ratio I_y/I_x as a function of gate voltage. (c) *IV* curves of the same device measured along **x** and **y** directions at different gate voltages.

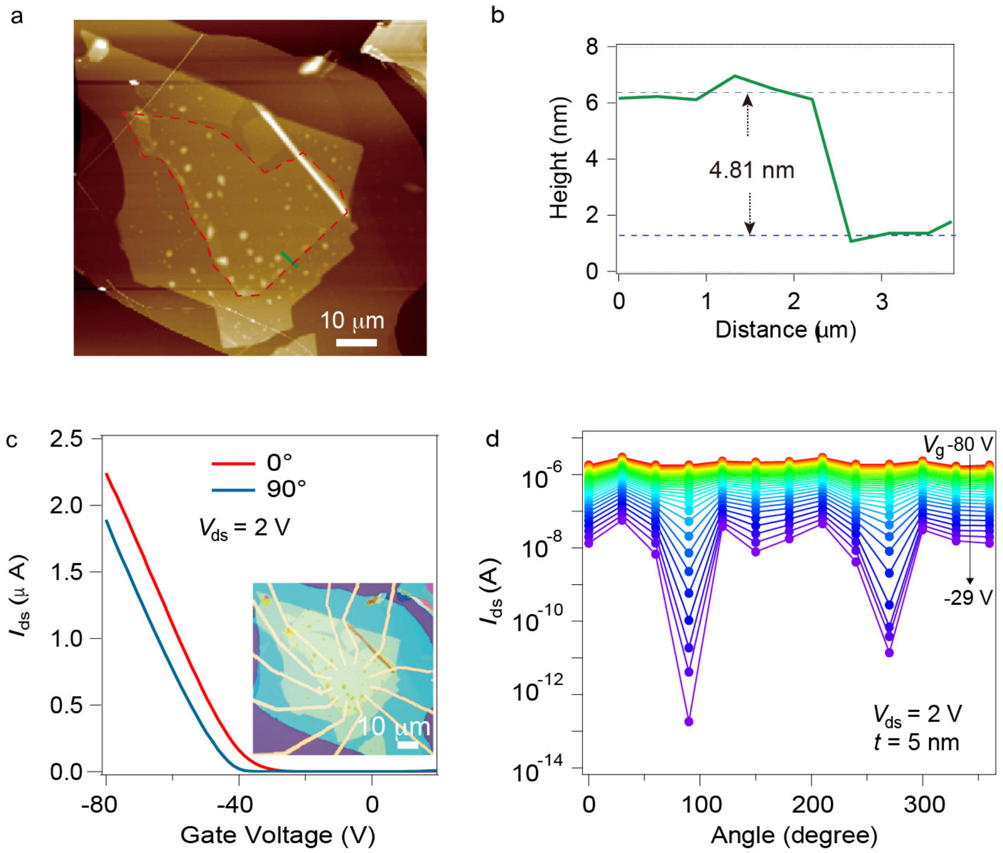

Supplementary Figure 24. GAR effect in 6-layered GaTe encapsulated by h-BN. (a) AFM scan of the h-BN/4.81nm GaTe/h-BN heterostructure, with the height profile along the solid green line plotted in (b). (c) Field effect curves along 0 (**y**-axis) and 90 degree (**x**-axis), respectively. Inset shows the optical image of the device. (d) Anisotropic source-drain current for different gate voltages. As inter-layer distance of GaTe is about 0.8 nm, we can draw the conclusion that, down to 6 atomic layer limit, the observed giant anisotropic resistance prevails.

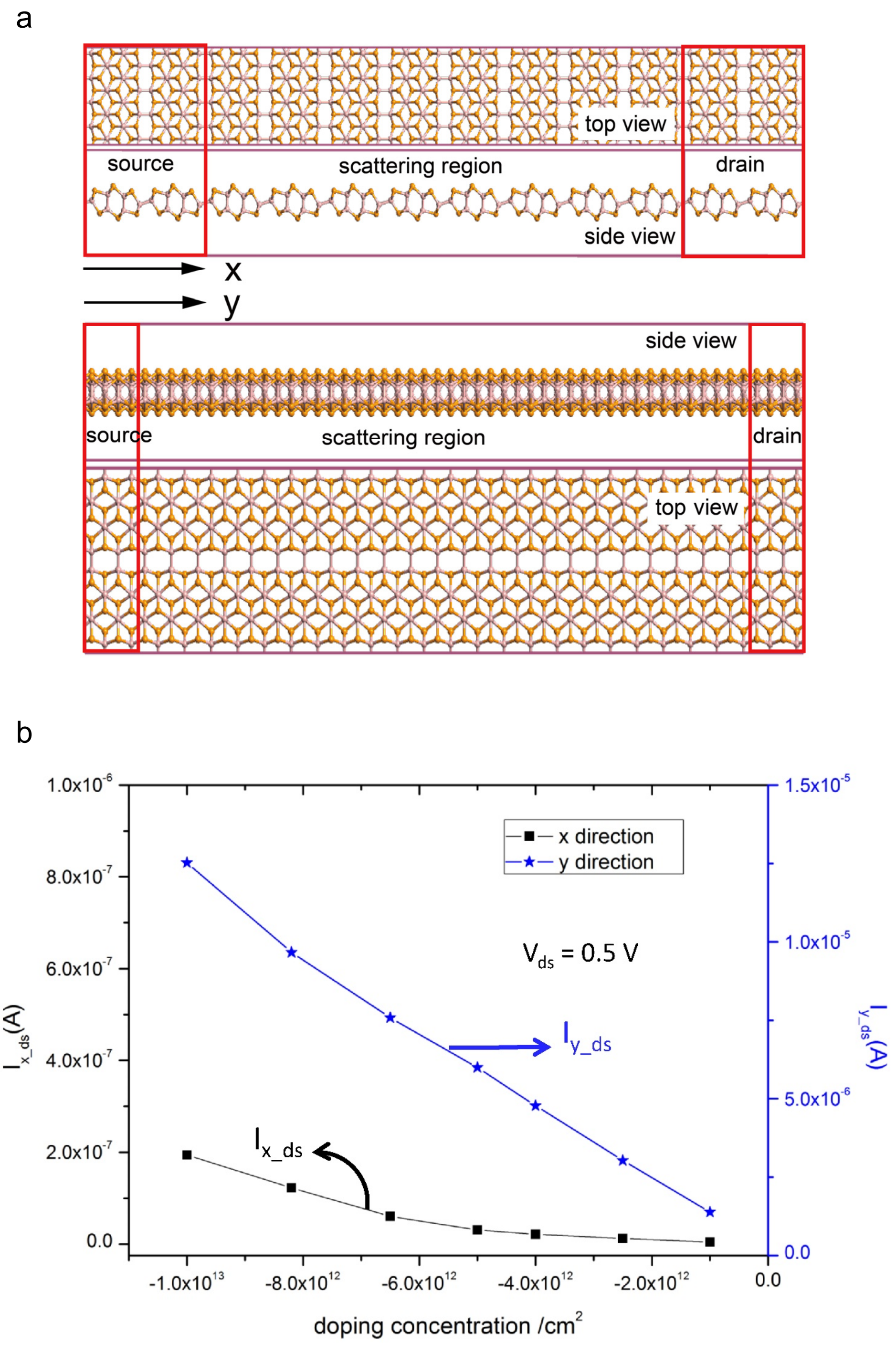

Supplementary Figure 25. (a) Device structure set up for both **x** and **y** directions. (b) The source-drain current I_{ds} as a function of carrier concentration calculated from non-equilibrium Green's function (NEGF) method at 0.5 V bias voltage for **x** (black square) and **y** (blue star) directions.

a

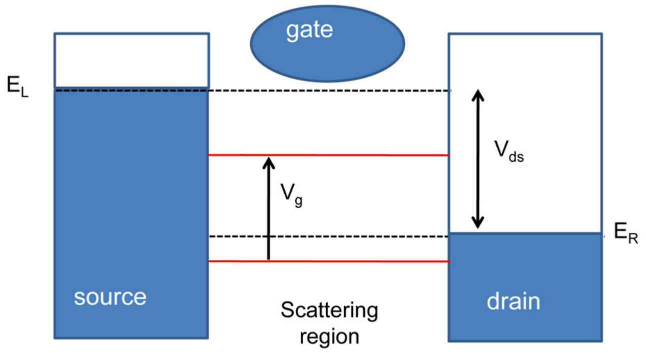

b

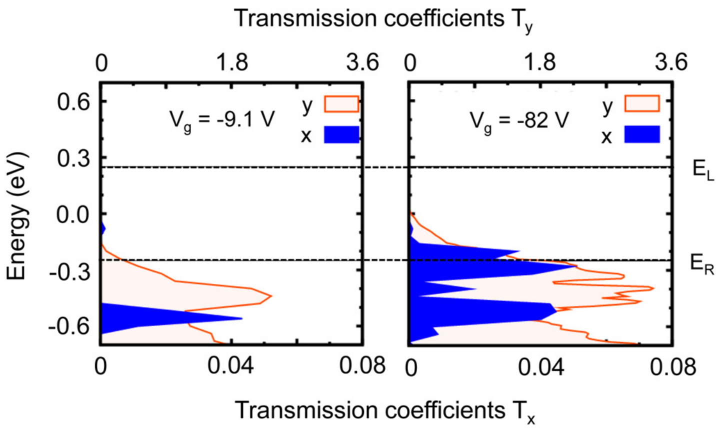

Supplementary Figure 26. (a) Energy profile of source, drain and gated scattering center region. (b) The transmission coefficients T_x and T_y at two gate voltages.

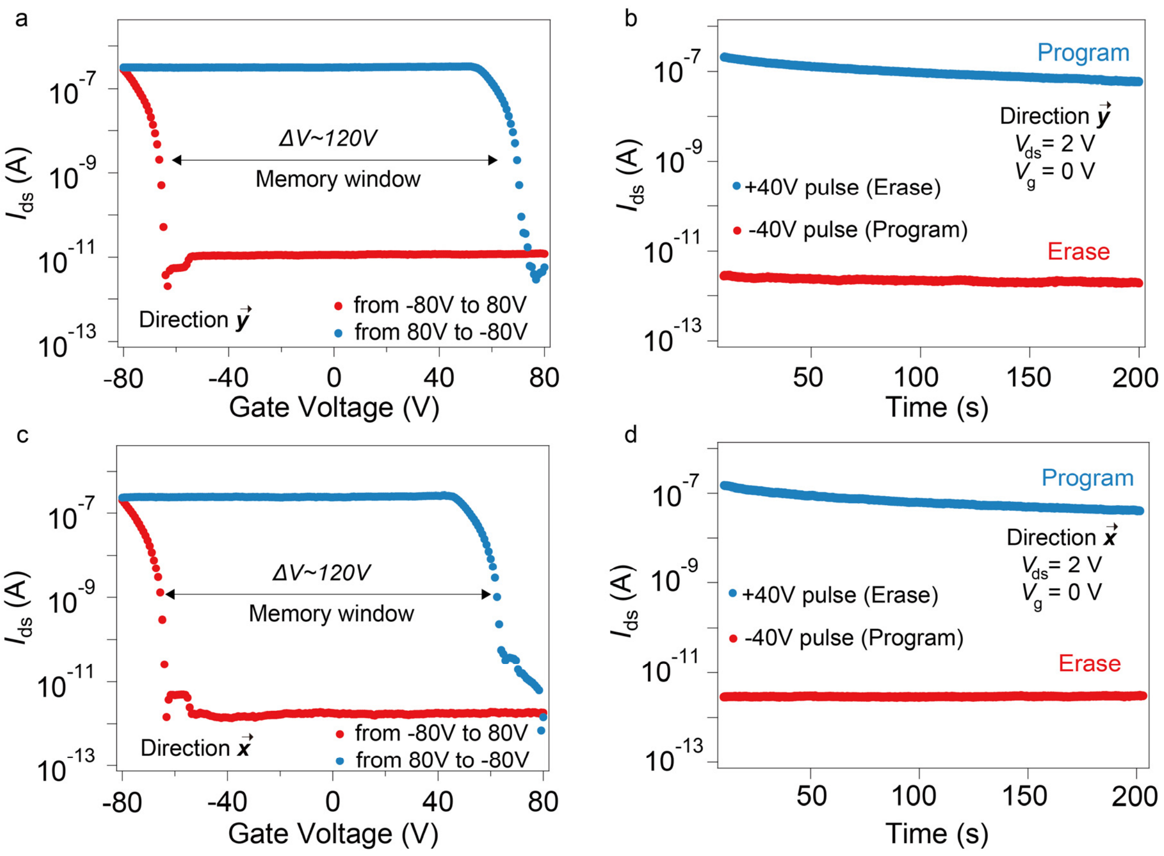

Supplementary Figure 27. (a),(c) Transfer characteristic of the floating gate device (sample-S6, same device illustrated in Fig. 3 in the main text) along ***y*** and ***x*** directions, respectively. The large hysteresis of ~120 V is related to accumulation of charge in the multilayer graphene floating gate. (b),(d) Temporal evolution of source drain currents in the erased (ON) and programmed (OFF) states along **y** and **x** directions, respectively.

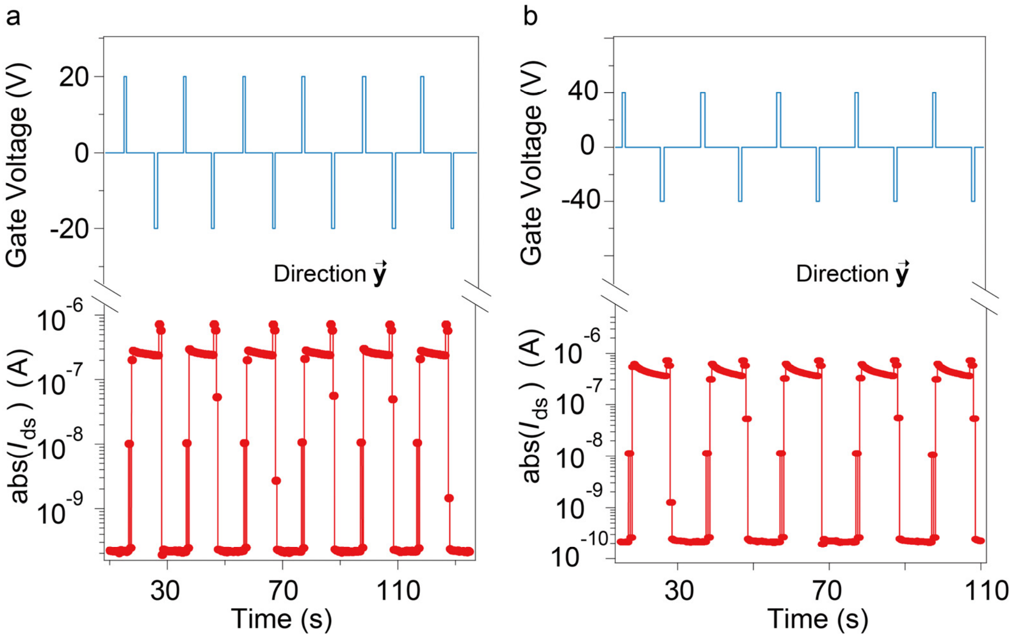

Supplementary Figure 28. Demonstration of erasing and programming pulses in memory-**y** at V_g=-20 V in (a), and -40 V in (b), respectively.

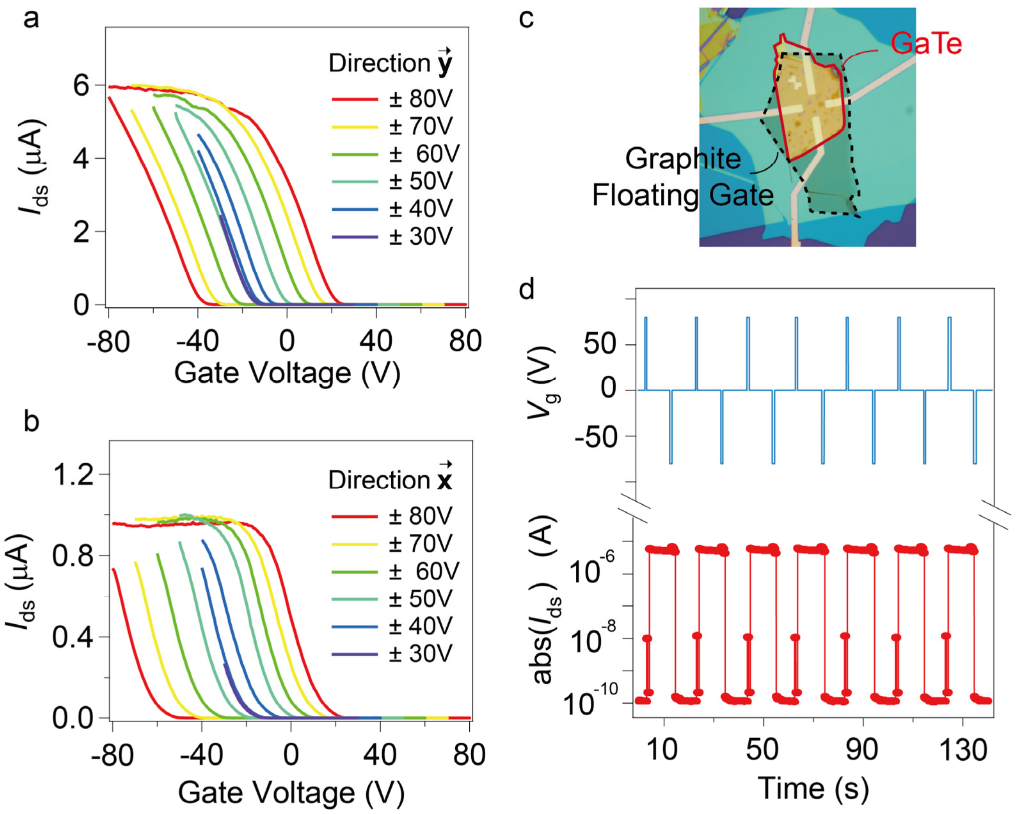

Supplementary Figure 29. (a),(b) Memory windows of device S4 (the device as that discussed in Fig.4 in the main text), along **y** and **x** directions, respectively. Optical image of the device is shown in (c). (d) Demonstration of erasing and programming pulses in memory-**y**.

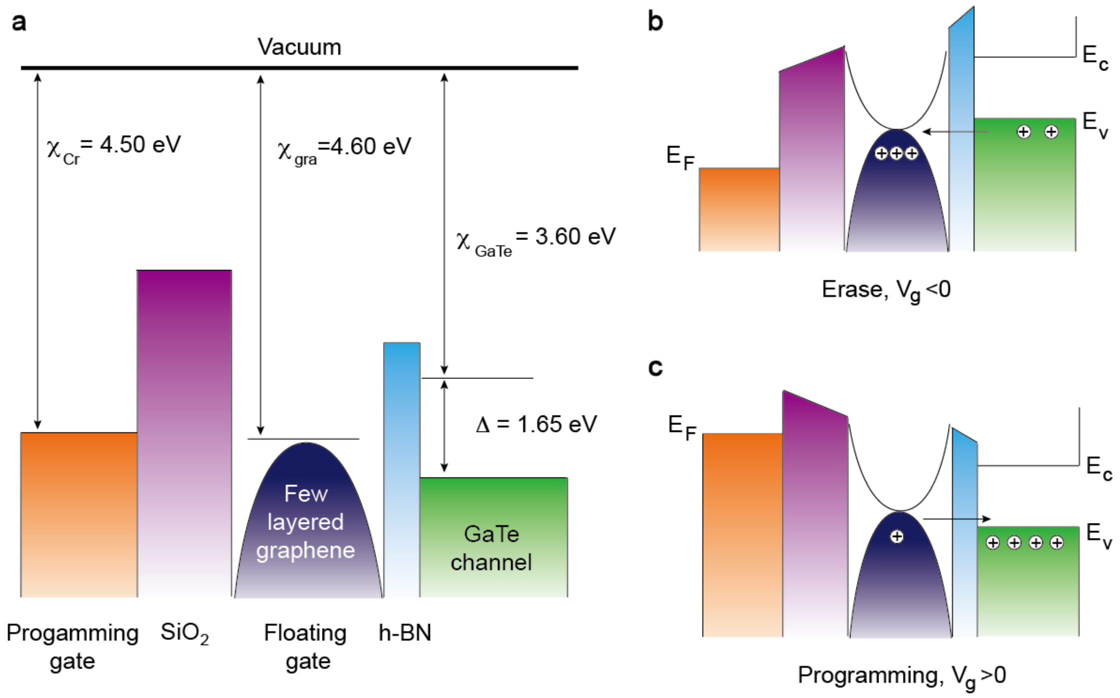

Supplementary Figure 30. Simplified band diagram of the GaTe floating gate memory devices. (a) Illustration of the electron affinities (χ) of different layers involved (Cr, SiO_2, Graphene, GaTe) in the heterostructures. (b) During the rease process, Fermi level of the programming gate (Au/Cr, with Cr as the adhesion layer during evaporation) is shifted down. And fewer holes are populated in the GaTe chanel, with the positive charges being accumulated in the floating gate, and vice-versa (c).

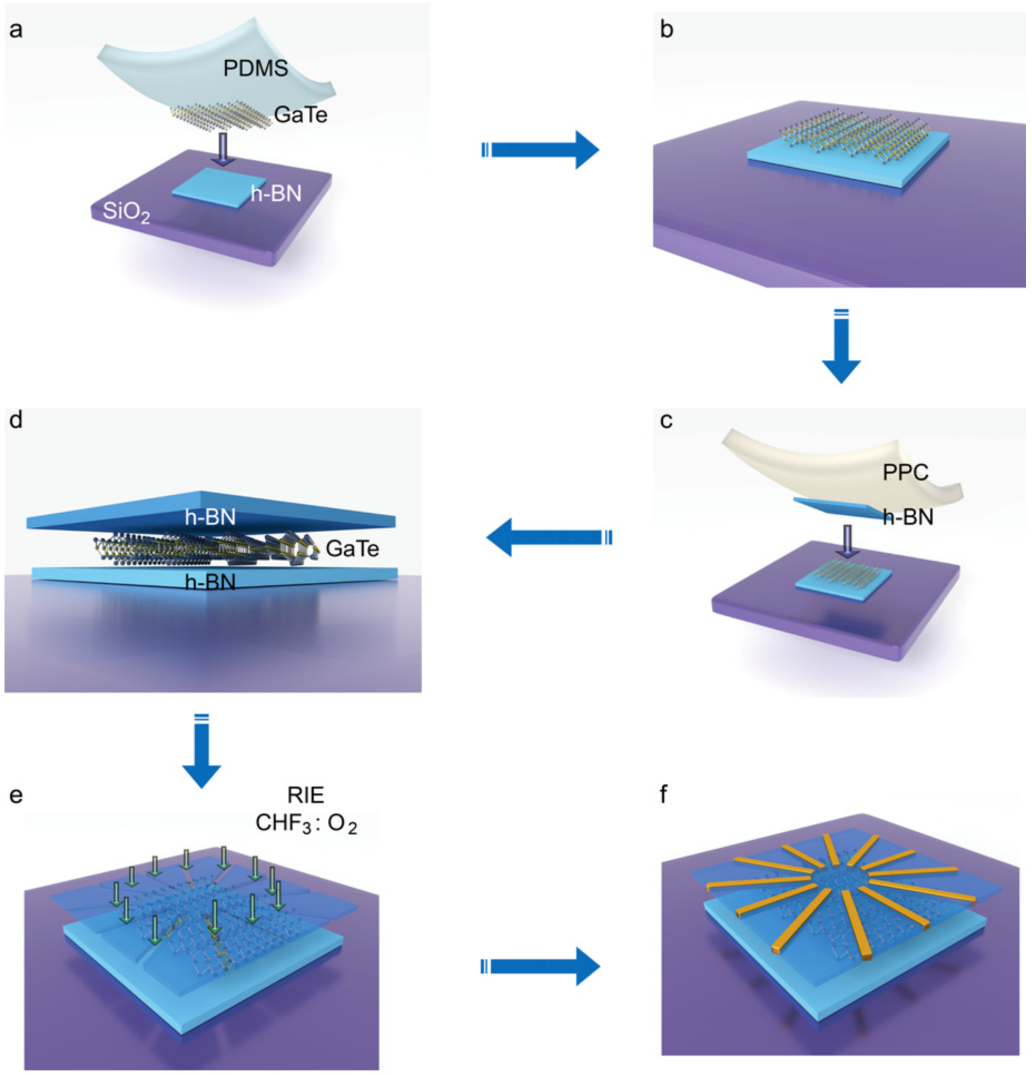

Supplementary Figure 31. Art view of the fabrication process of h-BN/GaTe/h-BN heterostructure device. (a) A few-layered GaTe flake is exfoliated on a PDMS substrate, and then is transferred on a selected h-BN which was exfoliated previously on a SiO_2/Si substrate. (b) After transfer, the GaTe is separated from PDMS by van der Waals force. (c) Another selected h-BN is picked up from the SiO_2/Si substrate by poly propylene carbonate (PPC), and then transferred onto the h-BN/GaTe heterostructure. (a)-(c) are done in a glove box. (d) The few-layered GaTe flake is encapsulated by two h-BN layers, then annealed at 320 ^{o}C to remove the PPC residues completely. (e) A dry etching is used to pattern the stack, with the top h-BN etched by reactive ion etching, making the GaTe flake partially exposed. (f) Twelve electrodes (Cr/Au) are fabricated by e-beam lithography and thermal evaporation metallization.

Supplemental Methods

PL was measured with a home-built scanning confocal system based on an invert microscope (Nikon, TE2000-U), equipped with a commercial CCD (Princeton Instrument, PIXIS), a 100× objective lens (numerical aperture: 0.9), and 1200 lines/mm gratings. The excitation wavelength was 532 nm from a semiconductor laser. All the experiments were performed at the room temperature. The polarized PL spectra were obtained by rotating the incident laser beam to change the angle, θ, between the crystal direction and the polarization of the incident laser beam. The zero-angle direction was defined by the experimental system as a reference at the beginning of the experiments.

Supplemental References

1. Fonseca, J. J. *et al.* Bandgap restructuring of the layered semiconductor gallium telluride in air. *Adv. Mater.* **28**, 6465-6470 (2016).

2. Wang, Z. *et al.* Role of Ga vacancy on a multilayer GaTe phototransistor. *ACS Nano* **5**, 4859-4865 (2014).

3. S. Huang, Y. Tatsumi, X. Ling, H. Guo, Z. Wang, G. Watson, A. A. Puretzky, D. B. Geohegan, J. Kong, J. Li, T. Yang, R. Saito and M. S. Dresselhaus, *ACS Nano*, 2016, **10**, 8964-8962.

4. X. Wang, A. M. Jones, K. L. Seyler, V. Tran, Y. Jia, H. Zhao, H. Wang, L. Yang, X. Xu and F. Xia, *Nature Nanotechnology*, 2015, **10**, 517-521.

5. D. Tan, H. E. Lim, F. Wang, N. B. Mohamed, S. Mouri, W. Zhang, Y. Miyauchi, M. Ohfuchi and K. Matsuda, *Nano Research*, 2016, **10**, 546-555.

6. H. Zhao, J. Wu, H. Zhong, Q. Guo, X. Wang, F. Xia, L. Yang, P. Tan and H. Wang, *Nano Research*, 2015, **8**, 3651-3661.

7. F. Xia, H. Wang and Y. Jia, *Nature Communications*, 2014, 5, 4458.
